# Elevated retrocopy burden and sloth-specific expansions illuminate mammalian genome evolution

**DOI:** 10.1186/s12915-026-02632-5

**Published:** 2026-05-19

**Authors:** Marcela Uliano-Silva, Helena Beatriz da Conceição, Rafael L. V. Mercuri, Sylke Winkler, Gabriela D. A. Guardia, Eugene Myers, Shane McCarthy, Alan Tracey, Alexander Suh, Mark Blaxter, Pedro A. F. Galante, Camila J. Mazzoni

**Affiliations:** 1 Tree of Life, Wellcome Sanger Institute, Cambridge, UK; 2 Faculty of Biosciences and Aquaculture, Nord University, Bodø, Norway; 3 Molecular Oncology Center, Hospital Sírio Libanês, São Paulo, Brazil; 4 The Institute of Mathematics and Statistics of the University of São Paulo, São Paulo, Brazil; 5 Max-Planck Institute for Molecular Cell Biology & Genetics, Dresden, Germany; 6 Centre for Molecular Biodiversity Research, Leibniz Institute for the Analysis of Biodiversity Change, Museum Koenig Bonn, Adenauerallee 160, Bonn, 53113 Germany; 7 Bonn Institute for Organismic Biology (BIOB)–Animal Biodiversity, University of Bonn, Bonn, Germany; 8 Department of Evolutionary Genetics, Leibniz Institute for Zoo and Wildlife Research (IZW), Berlin, Germany; 9 Berlin Center for Genomics in Biodiversity Research (Begendiv), Berlin, Germany

**Keywords:** Retrocopy, LINE1 retrotransposon, Retrogene domestication, Comparative genomics, Genome evolution, Metabolic adaptation, Sloths, Xenarthra, Mammals

## Abstract

**Background:**

Xenarthrans, comprising sloths, anteaters, and armadillos, represent one of the most morphologically and physiologically specialized mammalian clades, yet the genomic basis of their adaptations remains poorly understood. Here, we present chromosome-level genomes for the two-toed sloth (*Choloepus didactylus*) and the southern anteater (Tamandua tetradactyla) and investigate how retrotransposon-mediated gene duplications (retrocopies) have shaped genome evolution in these and other species in Xenarthra.

**Results:**

Comparative analyses revealed that the xenarthran genomes analyzed here harbour the highest number of retrocopies reported among mammals, with lineage-specific insertion dynamics. Anteater and armadillo genomes contain older LINE1 repertoires and species-specific older retrocopy insertions. In contrast, sloths retain both an abundance of young LINE1s and thousands of young retrocopies, alongside a large shared set that originated from an evolutionary burst of retroduplication in the branch leading to their last common ancestor (~ 30 Mya). In *C. didactylus*, 49% of retrocopies were found to be expressed in five tissues, compared with 27% in *Dasypus novemcinctus* in three tissues. Evolutionary analyses identified 38 retrocopies with strong hallmarks of domestication in *C. didactylus*. Many of these retrocopies derive from parental genes involved in mitochondrial and metabolic processes, suggesting a potential genomic contribution to the physiological specializations of sloths.

**Conclusions:**

Altogether, our findings identify retrotransposition as a major contributor to the genomic architecture of the xenarthrans presented here and highlight retrocopy origination as a mechanism for generating lineage-specific novelty and, possibly, distinctive biological specializations.

**Supplementary Information:**

The online version contains supplementary material available at 10.1186/s12915-026-02632-5.

## Background

Sloths, anteaters, and armadillos comprise the superorder Xenarthra, one of the four deep-branching clades of placental mammals and the only one to have originated in South America [1, 2]. They arose in the Late Paleocene (approximately 60–70 Mya) and once included hundreds of species, from giant ground sloths to glyptodonts and pampatheres, but following terminal Pleistocene mass extinctions, only 43 species remain [3], distributed in two orders: Cingulata (armadillos) and Pilosa (anteaters and sloths). Xenarthrans display striking morphological and physiological adaptations. Armadillos are the only extant mammals with articulated osteoderms, forming a protective carapace, and have robust, clawed forelimbs adapted for digging and, in some species, conglobation [4, 5]. Pilosa includes anteaters, obligatory myrmecophagous with elongated rostra, vestigial or absent teeth, and long, protrusible tongues for feeding on social insects, and sloths (suborder Folivora). Modern tree sloths (*Choloepus and Bradypus*) are the only obligatory suspensory quadrupeds among mammals, with elongated limbs, curved claws, and specialized musculature that enable energy-efficient, inverted arboreal locomotion [6]. They are also exceptional in their deviation from the typical mammalian “rule of seven” cervical vertebrae [7]: *Choloepus* usually have 5–7, while *Bradypus* have 8–10. Sloths exhibit the lowest metabolic rates recorded among mammals, often less than half of what is expected for their body size, and show reduced muscle mass, slow digestion, and heterothermy, with body temperatures fluctuating around 5 °C [8, 9]. These metabolic adaptations, coupled with their distinctive forelimb morphology, permit a finely tuned and maximally slothful life.

Available xenarthran genomes have limited assembly contiguity, hampering large-scale comparative studies and leaving much of their nuclear genome evolution unexplored, particularly the repetitive elements that comprise substantial portions of mammalian genomes and can be key drivers of genetic innovations. Within this repeat landscape are found retrocopies, gene duplicates generated by the reinsertion of reverse-transcribed copies of spliced, polyadenylated mRNA into the genome [10]. In mammals, retrocopies arise when machinery derived from Long Interspersed Nuclear Element 1 (LINE-1) copies these mRNAs and integrates them via target-primed reverse transcription (TPRT) [11]. The resulting sequences are intronless, often 5′-truncated, and typically lack parental regulatory elements, characteristics that lead some authors to classify these gene duplicates as processed pseudogenes. However, even though some retrocopies are not expressed and become pseudogenes, others are “domesticated.” These often show positive selection [10], are expressed, and can have novel functions as tissue-specific paralogues [12] or as regulatory noncoding RNAs [13]. These diverse fates mean that retrocopies are both markers of genome history and potential contributors to lineage-specific biological innovations.

Here we present chromosome-level assemblies for the two-toed sloth (*Choloepus didactylus*) and the southern anteater (*Tamandua tetradactyla*), expanding genomic resources for Xenarthra. Using a standardized annotation pipeline, we reveal that xenarthrans harbour the highest number of retrocopies reported in any mammalian group to date, with dynamics that are lineage-specific and closely tied to their LINE1 evolutionary history. In sloths, in addition to a burst of young LINE1s and retrocopies, we identified retrocopies orthologous only within sloths, among which we identified candidates for domestication. Notably, many of these retrocopies are derived from mitochondrial and other metabolic-associated genes, suggesting that these retrotransposition events may contribute to the extreme metabolic adaptations of sloths. Our findings highlight retrotransposition and retrocopies as central forces shaping genome evolution in these xenarthran species, providing a framework for understanding how their dynamics can facilitate the evolution of extreme phenotypes. A Portuguese translation of the abstract is provided in Additional file 1.

## Results

### Two new high quality genomes for Xenarthra

We generated genome assemblies for *Choloepus didactylus* and *Tamandua tetradactyla* using the Vertebrate Genomes Project (VGP) v1.6 Assembly Pipeline (see Methods). Both genomes were sequenced using PacBio CLR long reads, scaffolded with Bionano optical maps, chromatin conformation capture (Hi-C) data, and manually curated to achieve high-quality, chromosome-level assemblies. The final assemblies have scaffold N50s of 146 Mb and 174 Mb, respectively (Table 1). The X and Y sex chromosomes were reconstructed in both assemblies. The large majority of the assembled sequences were assigned to chromosomes in both assemblies (99.94% in *C. didactylus* and 99.51% in *T. tetradactyla*; Figure S1).

**Table 1:** General metrics of *Choloepus didactylus* and *Tamandua tetradactyla* genome assemblies

Species	*Choloepus didactylus*	*Tamandua tetradactyla*
Accession	GCF_015220235.1	GCA_023851605.1
Total length	3,214,702,648	3,205,741,135
Scaffold N50	146,178,362	174,634,624
Contig N50	20,994,632	9,869,167
Scaffold count*	146	201
BUSCO** mammalian_odb10	C:95.7%[S:89.6%,D:6.1%],F:0.9%,M:3.4%,n:9226	C:96.1%[S:91.4%,D:4.7%],F:0.8%,M:3.1%,n:9226

^*^Including the mitochondrial genome as a scaffold

^**^BUSCO: Benchmarking using single-copy orthologues

**Fig. 1:**
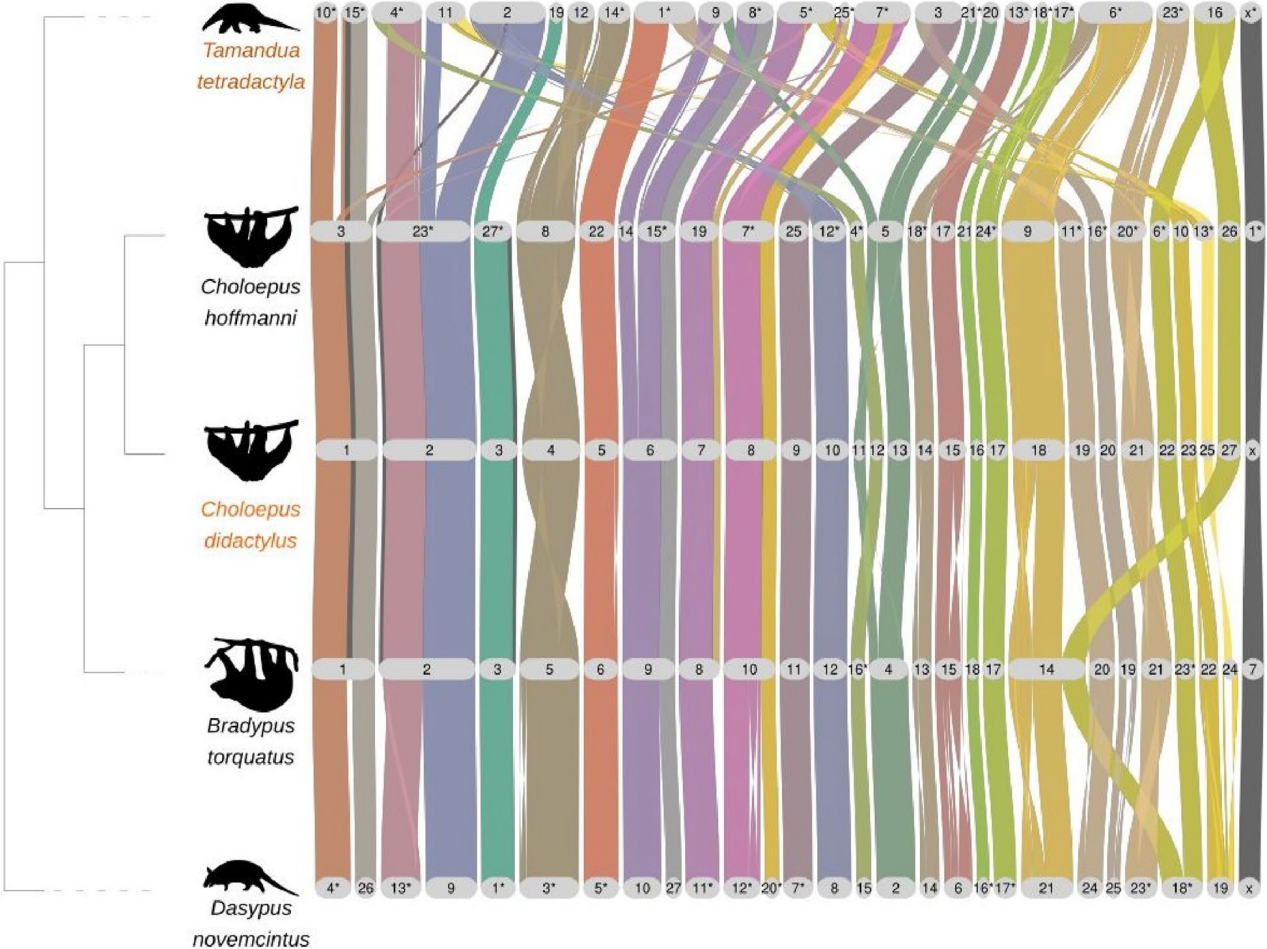
BUSCO-based synteny across xenarthran genomes. BUSCO-predicted genes and conserved syntenic blocks are shown for five Xenarthra genomes, visualized using GENESPACE [16]. The species highlighted in orange (*Choloepus didactylus* and *Tamandua tetradactyla*) are genome assemblies generated in this study. In each species, grey lozenges indicate assembled chromosomal pseudomolecules. Coloured ribbons connect orthologous blocks between species, asterisks represent flipped orientation. The cladogram on the left indicates phylogenetic relationships among the species

To investigate chromosome structure and genome-wide synteny in Xenarthra, we performed synteny analysis using Benchmarking Universal Single-Copy Orthologs (BUSCO [14]) orthologues across five species: the two newly sequenced genomes (*C. didactylus* and *T. tetradactyla*) and three publicly available genomes from the sloths *Bradypus torquatus* [15] and *Choloepus hoffmanni*, and the armadillo *Dasypus novemcinctus* (Fig. 1, Table S1). The analysis reveals conserved chromosome architecture in Xenarthra. The karyotype of *T. tetradactyla* appears to be the most divergent among the five species. In contrast, the three sloths and *D. novemcinctus* share more similar karyotypes, suggesting greater chromosomal stability in these lineages (Fig. 1).

### Recent LINE1 activity distinguishes sloth genomes within Xenarthra

**Fig. 2:**
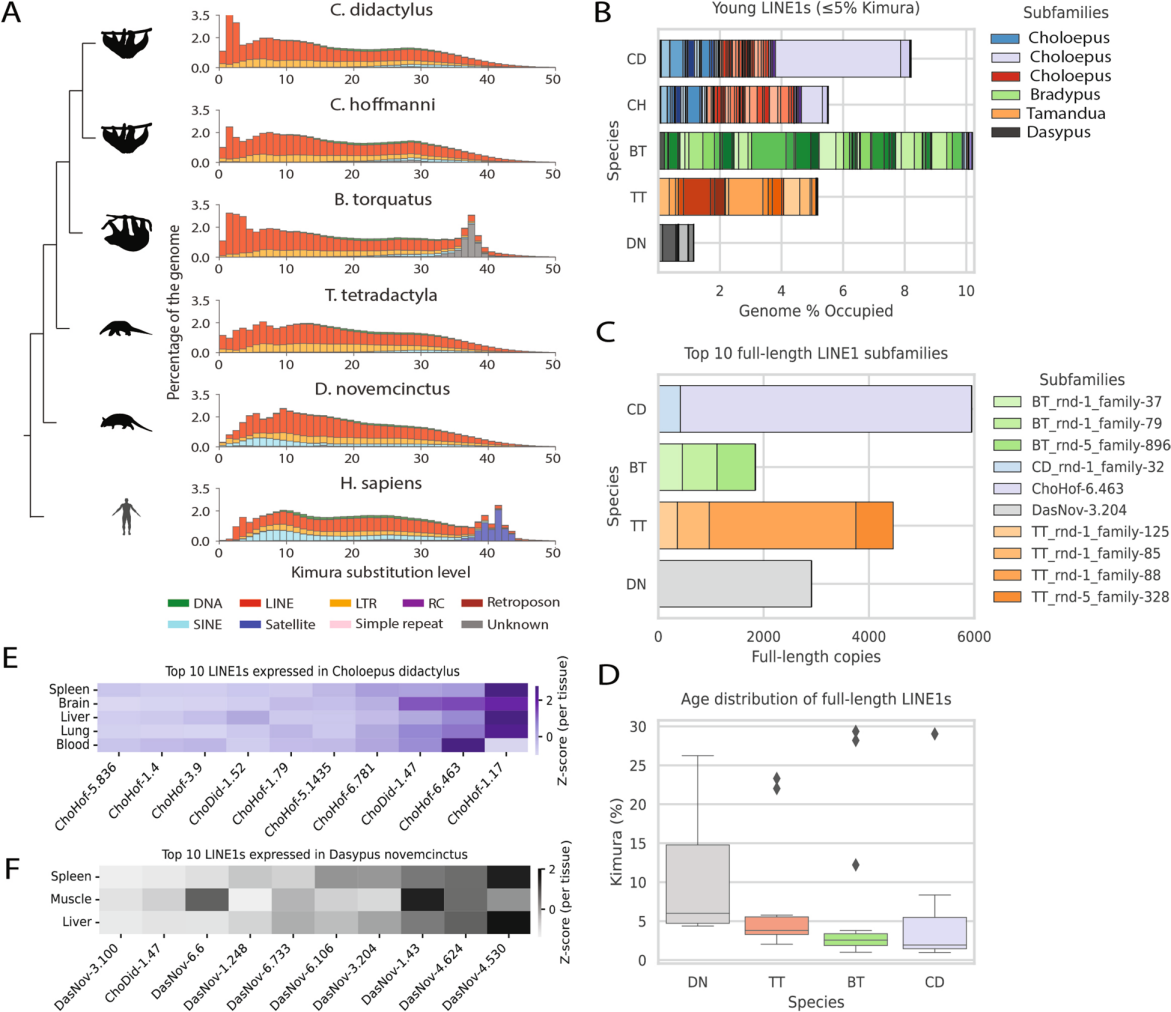
Repeat landscapes in xenarthrans and humans. **A** Kimura-corrected repeat landscapes for xenarthran and human genomes aligned to the species cladogram. **B** Proportion of the genome occupied by LINE1 subfamilies. **C** Total counts of LINE1 full-length copies for the 10 most abundant LINE1 subfamilies. **D** Mean Kimura divergence of all LINE1s identified as full-length. **E**–**F** Expression of LINE1 subfamilies in* C. didactylus* (**E**) and *D. novemcinctus* (**F**). Species abbreviations: CD, *C. didactylus*; CH, *C. hoffmanni*; BT, *B. torquatus*; TT, *T. tetradactyla*; DN, *D. novemcinctus*

To investigate repeat landscape evolution in Xenarthra, we merged species-specific libraries into a common library and used this to annotate all genomes consistently (see Methods). As in most mammals, xenarthran genomes are dominated by LINEs (Fig. 2A), with LINE1 being the most abundant family (Figure S2). However, in contrast to humans and other xenarthrans, sloth genomes show pronounced peaks of LINEs with low divergence from the inferred consensus, indicating recent bursts of activity (Fig. 2A).

We examined these young LINE1s in more detail by quantifying the contribution to genome content of subfamilies with a Kimura divergence of ≤ 5% across species. Sloths have a larger proportion of their genomes occupied by young LINE1s compared with other xenarthrans, with *B. torquatus* showing the highest proportion (Fig. 2B). Independent 1 Mb window analyses confirmed that these insertions occupy significantly larger genomic fractions across sloth genomes (Mann–Whitney U test, *p* = 3.6 × 10⁻33). Within sloths, most LINE1 subfamilies are shared between *C. didactylus* and *C. hoffmann*i, consistent with their more recent divergence (~ 5 Mya) [17]. In contrast, *B. torquatus* shares only a subset with *Choloepus*, and most of its young LINE1s are species-specific, reflecting its earlier divergence within Folivora (~ 30 Mya) [17, 18]. Beyond sloths, no young LINE1 subfamilies (≤ 5% divergence) are shared between all sloths and *T. tetradactyla* or *D. novemcinctus*, both of which harbour mostly species-specific expansions.

We also identified thousands of full-length LINE1s in all xenarthran genomes, except for *C. hoffmanni*, where only nine were recovered (Fig. 2C). Given the fragmented nature of this assembly (Tables S1 and S2), we evaluated whether the apparent depletion of full-length LINE1s resulted from assembly limitations rather than biological loss by performing an assembly-independent k-mer analysis using raw sequencing reads (Methods; Figure S3; Table S3). k-mers from young LINE1s (≤ 5% divergence) were quantified and normalized to total read k-mers in counts per million (CPM). In *C. hoffmanni*, young LINE1 k-mers show strong enrichment relative to the broader LINE1 population (33.8-fold), comparable to enrichment levels in *C. didactylus* (34.7-fold) and *B. torquatus* (32.8-fold), whereas *T. tetradactyla* and *D. novemcinctus* exhibit lower enrichment (22.8-fold and 18.2-fold). These results indicate that young LINE1 sequence is abundant in *C. hoffmanni*, and that reduced recovery of full-length elements is likely due to assembly fragmentation or repeat collapse rather than true depletion.

In the long-read assemblies, the highest numbers of full-length LINE1s were observed in *C. didactylus* (6700 full-length copies), followed by *T. tetradactyla* (5159). In most species, expansions were driven by a single dominant, species-specific LINE1 subfamily (Fig. 2C). The subfamilies forming full-length LINE1s tend to be younger in sloths than in the anteater and armadillo (Fig. 2D). For instance, *C. didactylus* is dominated by 5531 full-length copies of subfamily ChoHof-6.463 (Kimura ~ 2%), while the most abundant family in *T. tetradactyla* is 2785 copies of TT_rnd-1_family-88 (Kimura ~ 2.4%), and in *D. novemcinctus* is 2910 copies of DasNov-3.204 (Kimura ~ 5.2%).

To assess possible LINE1 transcriptional activity, we analyzed RNA-seq data (see Methods) from *C. didactylus* and *D. novemcinctus* (Fig. 2E and F). Several LINE1 subfamilies showed detectable expression in both species. In *C. didactylus*, the young ChoHof-6.463 subfamily (Kimura ~ 2%, 5,531 full-length copies) had higher expression than most other LINE1s, with particularly elevated levels in blood tissue. In contrast, *D. novemcinctus* expressed DasNov-3.204 (Kimura ~ 5.2%, 2,910 full-length copies), but this was not among the most highly expressed LINE1s. Instead, its top-expressed elements (DasNov-4.530, DasNov-4.624, DasNov-1.43) all lack full-length representatives and have mean Kimura divergences > 7%, suggesting pervasive or readthrough transcription of fragments rather than autonomous LINE1 activity in *D. novemcinctus*.

These results indicate that each xenarthran species exhibits a distinct LINE1 dynamic, reflecting their evolutionary divergence. In sloths, however, several lines of evidence point to more recent and potentially ongoing LINE1 activity. These include the high genomic proportion of young LINE1s (Fig. 2A, B), the abundance of full-length elements and their younger age distribution (Fig. 2C, D), and the elevated expression of a recently expanded subfamily in *C. didactylus* (Fig. 2E). This contrasts with the older LINE1 expansions observed in *T. tetradactyla* and *D. novemcinctus*, where ancient insertions predominate and younger elements show small or non-detectable expression.

### Retrocopies are abundant in the studied xenarthran genomes

**Fig. 3:**
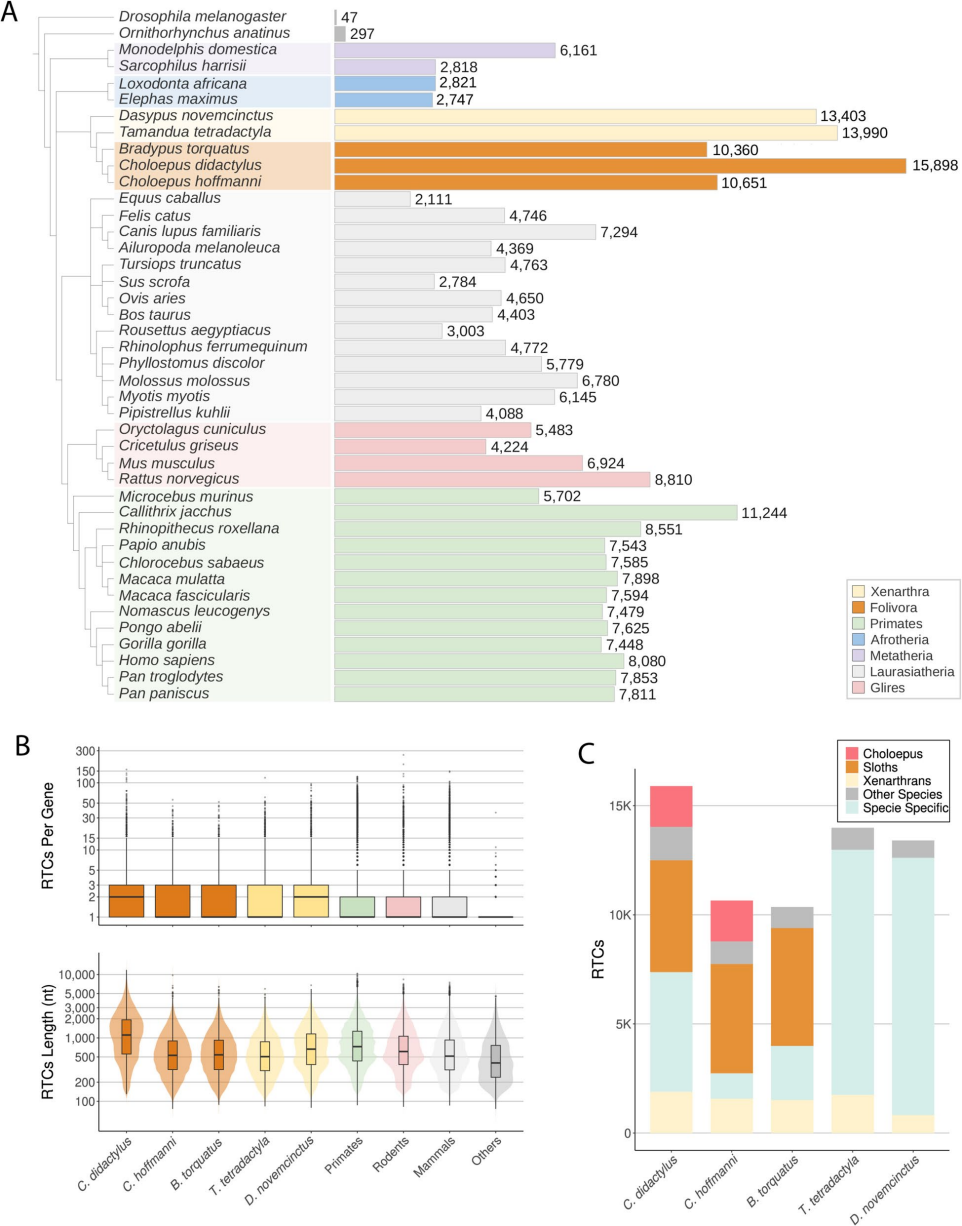
Xenarthran genomes harbour the highest number of retrocopies among mammals in our comparative dataset. **A** Total retrocopy (RTCs) counts across mammals and *Drosophila melanogaster*. Horizontal bars represent the number of retrocopies identified per species. The phylogenetic tree highlights Xenarthra in yellow and the suborder Folivora (sloths) in orange. **B** Retrocopy profiles. Upper panel: box plots showing the number of retrocopies per protein-coding parental gene. Lower panel: violin plots and boxplots showing retrocopy length distributions (in nucleotides). **C** Orthology relationships of retrocopies across Xenarthra. Bar heights represent total retrocopy counts per species, with colors indicating orthology groups. “Other species” represent retrocopies with orthology to other mammals

Because LINE1s provide the enzymatic machinery that drives retrocopy formation from protein-coding genes [11], we next investigated the abundance and evolutionary dynamics of retrocopies across Xenarthra. Using our standardized annotation pipeline [19], we catalogued retrocopies in all available xenarthran genomes, including our new *C. didactylus* and *T. tetradactyla* assemblies, and compared them to patterns across 41 mammalian species in our database (Fig. 3). We broadly define retrocopies as intronless, mRNA-derived inserts, regardless of sequence decay, and reserve the term “putatively domesticated” for the subset that, upon downstream analyses, exhibits hallmarks consistent with neofunctionalization.

The xenarthran genomes analyzed here harbour the highest retrocopy numbers, representing the largest retrocopy burden reported among mammals to date (Fig. 3A). We identified 13,403 retrocopies in *D. novemcinctus*, 13,990 in *T. tetradactyla*, 10,360 in B. torquatus, 10,651 in *C. hoffmanni*, and 15,898 in *C. didactylus*—the latter representing the largest retrocopy count reported for any mammal, or animal, so far. These counts far exceed those in other mammals, which typically range between 2000 and 8000 retrocopies per genome [19], indicating a pronounced expansion of retrocopies in the sampled xenarthran genomes (Fig. 3A). These results are robust to differences in gene annotation, as highly similar retrocopy counts are obtained using alternative annotation strategies (see Discussion).

To investigate the relationship between the elevated number of retrocopies and genome composition, we tested associations between retrocopy counts and genome size, number of protein-coding genes, and LINE1 abundance across species. Retrocopy counts showed weak association with gene number and moderate associations with both genome size and LINE1 abundance (Figure S4), with the strongest relationship observed for LINE1 content. Phylogenetically independent contrasts (PIC) analyses confirmed that these relationships remain significant after accounting for shared evolutionary history (Table S4). Across these relationships, xenarthran species consistently occupy the upper extreme of the distributions, likely driven by particularly high LINE1 activity (Figure S4).

The distribution of retrocopies per parental gene follows a consistent pattern across species (Fig. 3B, upper panel). In most mammals, parental genes typically generate a single retrocopy, with only a minority undergoing extensive retroduplication [19]. In *C. didactylus* and *D. novemcinctus*, however, the median is two retrocopies per parental gene, and the number of highly duplicated genes is particularly pronounced in *C. didactylus*, with some parental genes giving rise to more than 100 retrocopies. Statistical analysis of the distribution of retrocopy burden per parental gene (Table S5) shows that *C. didactylus* has significantly higher numbers of copies than other mammals (corrected *p* value < 0.0001), except for *D. novemcinctus* (corrected *p* value = 0.12). *C. didactylus* also exhibits a distinctive retrocopy length profile (Fig. 3B, lower panel). While most species show median retrocopy lengths of 500–750 nucleotides (nt), *C. didactylus* retrocopies are significantly longer, with a median of 1100 nt (Wilcoxon test, *p* < 0.0005).

Considering the much expanded number of retrocopies in Xenarthra, we next explored their evolutionary origins by extracting retrocopy sequences with flanking regions and aligning them across multiple mammalian genomes to assign orthology (see Methods). Only a small fraction (< 15% across all species) represent orthologous retrocopies shared among all xenarthrans (Fig. 3C). Most retrocopies in *T. tetradactyla* and *D. novemcinctus* were found to be species-specific, and may reflect independent insertion events at either species or clade levels (suborder Vermilingua and order Cingulata, respectively). With three representative species from both genera, we show that sloths retain a large shared subset: over half of retrocopies in B. torquatus (52%), and ~ 47% and 32% in *C. hoffmanni* and *C. didactylus*, respectively, are exclusive to sloths, consistent with origin in the branch leading to their last common ancestor ~ 30 Mya [17, 18], after their divergence from other xenarthrans. Additional retrocopies are shared only between *C. didactylus* and *C. hoffmanni*, consistent with retrotransposition activity in their more recent common ancestor (~ 5 Mya). Among sloths, *C. didactylus* shows the largest fraction of species-specific retrocopies (34%), followed by *B. torquatus* (23%) and *C. hoffmanni* (10%).

**Fig. 4:**
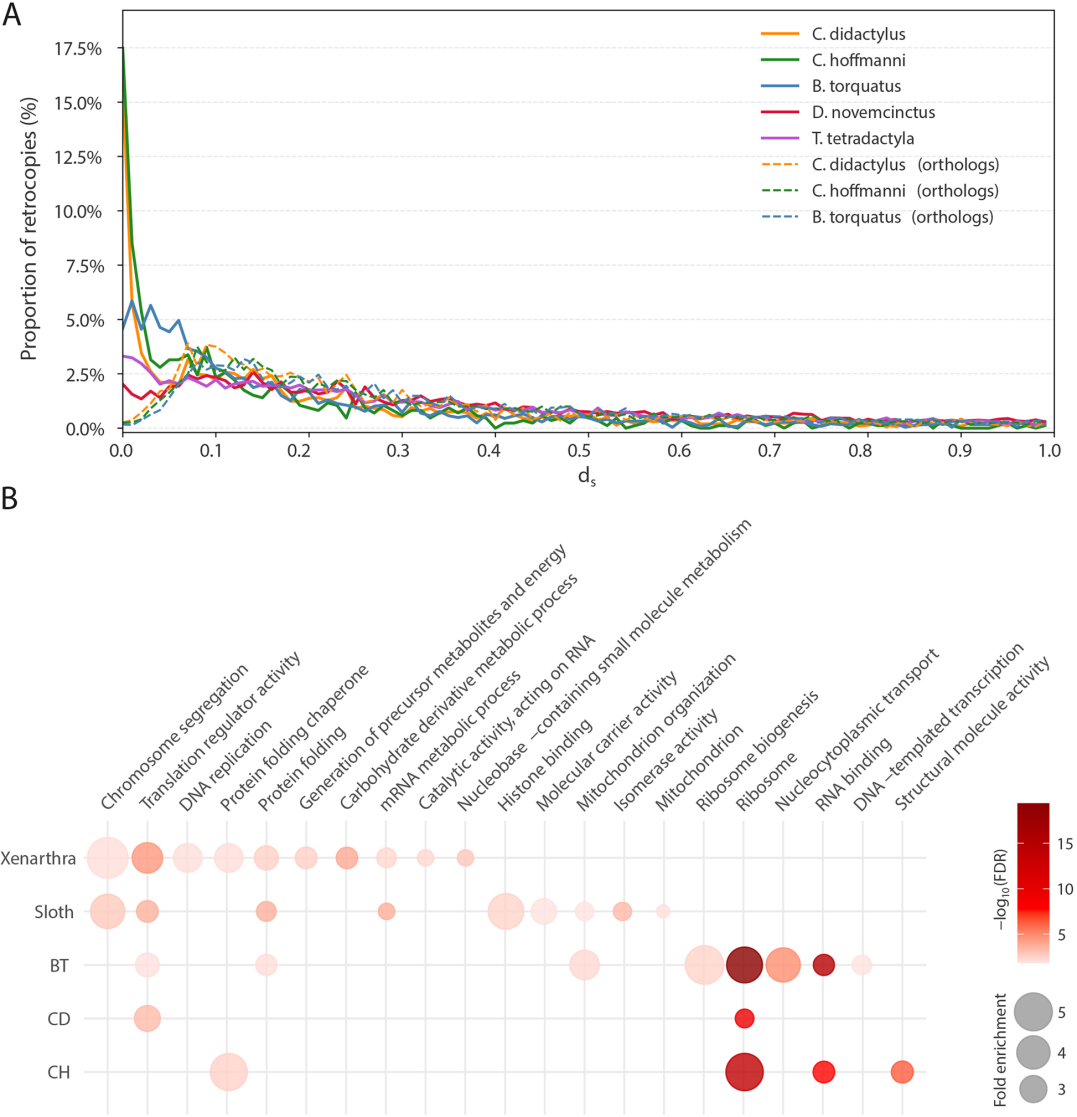
Evolution and function of retrocopies in sloths. **A** Distribution of synonymous substitution rates (dS) between retrocopies and their parental genes in *B. torquatus*, *C. hoffmanni*, *C. didactylus*, *T. tetradactyla*, and *D. novemcinctus*. Solid lines represent all species-specific retrocopies; dashed lines indicate only those found to be orthologous across the three sloth species. Peaks at low dS values, particularly in *C. didactylus*, *C. hoffmanni* suggest a larger proportion of very recent or preserved retrocopies. **B** Gene Ontology enrichment of parental genes giving rise to retrocopies in Xenarthra, sloths, or individual sloth species. Background sets included all parental genes with GO annotations from the respective species or lineage. Species abbreviations: CD, *C. didactylus*; CH, *C. hoffmanni*; BT, *B. torquatus*

In addition, to investigate retrocopy age and evolutionary trajectories, we predicted open reading frames (ORFs) and estimated synonymous substitution rates (dS) from codon-aware alignments of retrocopy–parental gene pairs. We then compared dS distributions for species-specific retrocopies and sloth-only orthologs (Fig. 4A). As expected, sloth-only retrocopies show no peak near dS = 0, consistent with insertion events predating the divergence of sloths (~ 30 Mya) and subsequent sequence decay. By contrast, species-specific retrocopies reveal distinct lineage-specific signatures: in sloths, a substantial fraction of retrocopies fall at dS ≈ 0 (17.6% in *C. hoffmanni*, 15.8% in *C. didactylus*, and 4.6% in *B. torquatus*), indicating recent or ongoing retrotransposition. Reflecting this pattern, sloth genomes are significantly enriched for very young retrocopies (dS ≤ 0.02) relative to the other xenarthrans analyzed (Fisher’s exact test, *p* = 4.9 × 10⁻10). In *T. tetradactyla* and *D. novemcinctus*, no equivalent peak at dS ≈ 0 is observed (Fig. 4A), suggesting that their bursts of retrocopy formation occurred earlier and that subsequent divergence has eroded retrocopy sequences.

Additional analyses of retrocopy-predicted ORFs further highlight these differences, revealing a greater proportion of young and potentially coding retrocopies in *C. didactylus*. This species exhibits the highest fraction of retrocopies retaining ORFs (75.6%), compared with *B. torquatus* (63.2%), *C. hoffmanni* (62.3%), and less than 60% in *T. tetradactyla* and *D. novemcinctus* (Figure S5A). Pairwise proportion tests indicate that ORF retention in *C. didactylus* is significantly higher than in all other species analyzed (*p* < 0.0001). *C. didactylus* also harbours the largest absolute number of ORFs (Figure S5B). While ORF length distributions are broadly similar across species (medians around 98 amino acids), *C. didactylus* shows a marked skew toward longer ORFs (Figure S5C, Wilcoxon rank-sum test, *p* < 0.001), consistent with more recent insertions and/or improved preservation of coding potential.

Taking these observations together, sloths stand out within Xenarthra for harbouring both an enrichment of full-length, low-divergence LINE1 families and peaks of young retrocopies (dS ≈ 0). In contrast, *D. novemcinctus* and *T. tetradactyla* show marks of older LINE1 repertoires and species-specific retrocopies that do not show comparable young dS peaks. These contrasting patterns suggest that retrocopy dynamics in these xenarthrans have been strongly shaped by lineage-specific differences in LINE1 activity.

### Functional annotation of parental genes yielding retrocopies

Next, we performed GO enrichment analysis of parental genes to identify functional biases in retrotransposition. Across the five xenarthran species, we found significant overrepresentation of terms related to fundamental cellular processes, with notable differences between major lineages (Fig. 4B). Overall, Xenarthra showed a broad range of enriched terms, including carbohydrate metabolism, protein binding, and various catalytic activities. As a group, the three sloth species (*B. torquatus*, *C. didactylus*, and *C. hoffmanni*) exhibited both shared and distinct enrichment patterns. All sloths showed enrichment in mitochondrial-related terms, including mitochondrial organization and mitochondrial activity, indicating a common bias toward retrotransposition of genes involved in energy metabolism. Notably, species-specific differences were also apparent. While *B. torquatus* displayed the most substantial enrichment for mitochondrial functions and ribosome biogenesis, *C. didactylus* and *C. hoffmanni* showed additional enrichment in cell cycle-related processes such as chromosome segregation, translation regulation, and DNA replication. The two-toed sloth *C. hoffmanni* further exhibited enrichment in nucleobase-containing small molecule metabolism and RNA binding, processes typically involving nucleotide metabolism and RNA processing. Together, these GO biological process enrichment analyses demonstrate both shared patterns across xenarthran lineages and lineage-specific biases. In particular, sloth species show notable enrichment for mitochondrial and energy-related functions.

### Retrocopy RNA-seq expression in *Choloepus didactylus* and *Dasypus novemcinctus*

Since some retrocopies may have high sequence similarity with their parental genes, especially recently formed ones, their expression quantification is susceptible to read misassignment and false positives. To mitigate this, we implemented a simulation-based filtering strategy (see Methods) to exclude retrocopy expression that could not be reliably distinguished from its parental gene (528 retrocopies were excluded for *C. didactylus* and 248 for *D. novemcinctus*). In addition, for highly similar retrocopies from the same parental gene, we aggregated their expression. We note that these aggregated values represent combined signals from similar retrocopies and may overestimate the number of independently expressed loci.

**Fig. 5:**
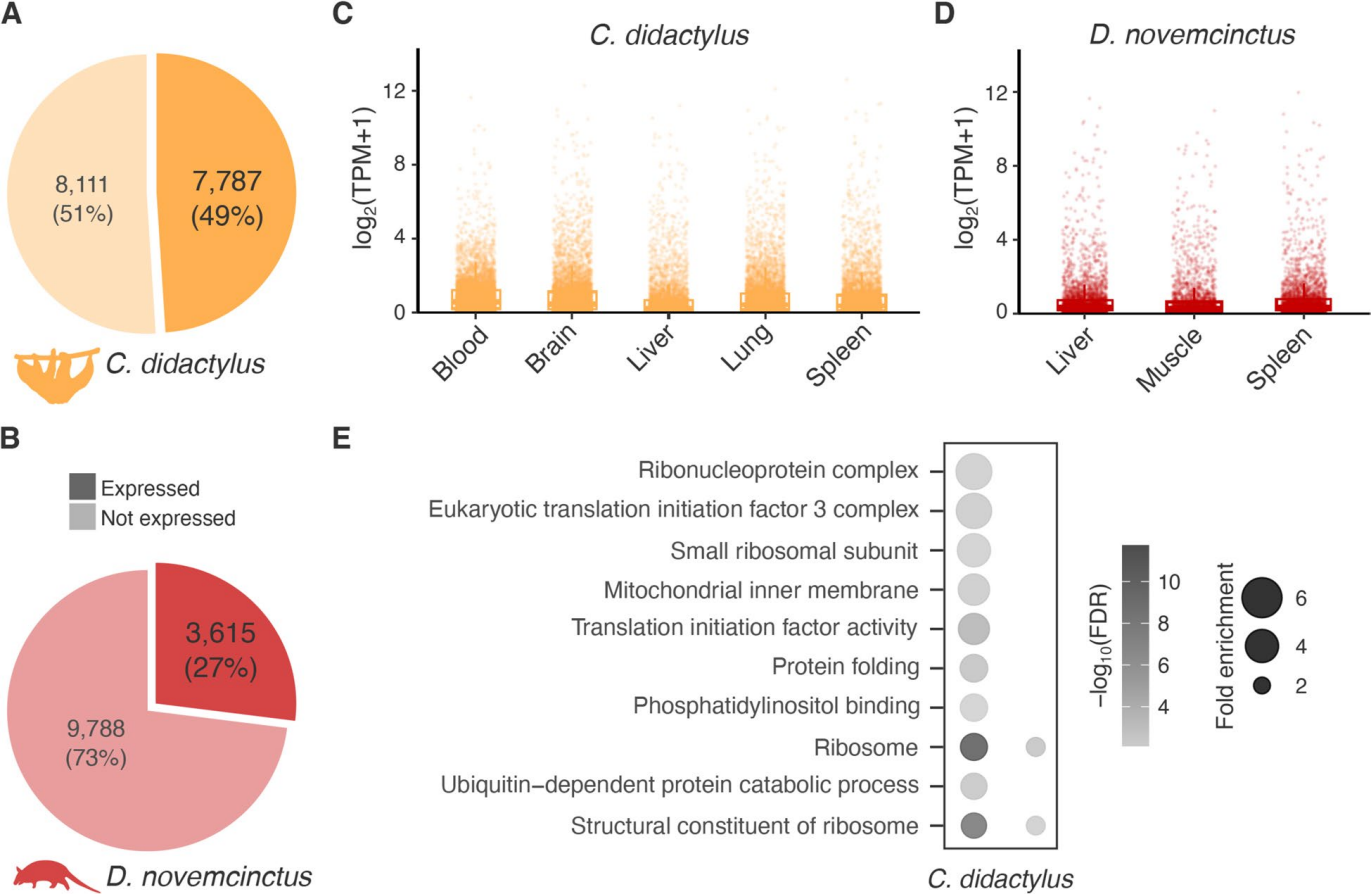
Retrocopy expression landscape in *C. didactylus* and *D. novemcinctus*. **A**–**B** Proportion of expressed (dark shading) retrocopies in *C. didactylus* and *D. novemcinctus*. **C**–D Distribution of retrocopy expression levels across tissues in *C. didactylus* and *D. novemcinctus*. **E** Gene Ontology enrichment analysis of the parental genes of expressed retrocopies in *C. didactylus*. The left panel shows enrichment using all protein-coding genes as background, whereas the right panel uses as background only the set of parental genes giving rise to retrocopies in this species. Only enriched terms (FDR < 0.01) are shown

We quantified retrocopy expression in five tissues of *C. didactylus* (spleen, brain, liver, lung, and blood) and three tissues of *D. novemcinctus* (spleen, muscle, and liver). Using this approach, we found expression for 7787 (49%) retrocopies in *C. didactylus* and 3615 (27%) retrocopies in *D. novemcinctus* (Fig. 5A–B) and a similar expression profile across tissues within each species (Fig. 5C–D). These results provide an overview of retrocopy transcriptional activity in each species based on the available datasets.

Because these RNA-seq datasets were generated for genome annotation purposes, rather than for controlled cross-species comparisons, the tissue panels differ between species. To assess whether differences in tissue composition could influence the proportion of expressed retrocopies observed in each species, we repeated the analysis using only the tissues sampled in both species (liver and spleen). This yielded consistent results, with *C. didactylus* still showing a higher proportion of expressed retrocopies compared to *D. novemcinctus* (χ2 = 26.76, *p* = 2.3 × 10⁻7). In both species, retrocopies presented consistently lower expression levels than their parental genes (Figure S6), as previously described for other mammals [12, 20]. Next, we performed a Gene Ontology enrichment analysis on the parental genes of expressed retrocopies in *C. didactylus*. This species presented significant overrepresentation of terms associated with fundamental cellular processes, including ribonucleoprotein complex, ribosome, structural constituent of ribosome, and translation-related functions (Fig. 5E, left), when using all protein-coding genes as background. When the analysis was repeated using as background only the parental genes giving rise to retrocopies, enrichment remained significant for ribosome-related categories, specifically ribosome and structural constituent of ribosome (Fig. 5E, right). These results suggest that retrotransposition has preferentially targeted genes involved in protein synthesis machinery in this species. We also performed analysis of synonymous substitution rates (dS) from ORF–parental gene alignments, which indicated that expressed retrocopies span a broad range of insertion ages (Figure S7). Notably, a significantly higher proportion of expressed retrocopies in *C. didactylus* falls within low dS values relative to those in *D. novemcinctus*. This pattern is significant in the full dataset (Fisher’s exact test, dS < 0.2: *p* < 3.4 × 10⁻9, OR = 1.34) and remains significant when restricting to shared tissues (*p* = 3.0 × 10⁻10, OR = 1.44; Figure S7).

### In search of function: potentially domesticated retrocopies in *C. didactylus*

To investigate whether some retrocopies have been retained under functional constraint (i.e., domesticated), we focused on *C. didactylus*. We refer to retrocopy domestication as the evolutionary process by which initially non-functional retrocopy insertions acquire biological function and are subsequently maintained by purifying selection, as evidenced by reduced nonsynonymous substitution rates and sustained expression patterns. A common approach to infer functional constraint is to examine the ratio of nonsynonymous to synonymous substitutions (dN/dS) between retrocopies and their parental genes. However, for very recent insertions, high sequence identity can produce artificially low dN/dS values that primarily reflect recency rather than genuine purifying selection. The presence of retrocopies with orthologs only across sloths (i.e., originated somewhere in the branch leading to their last common ancestor ~ 30 Mya) provides a valuable opportunity to overcome this bias and assess whether some retrocopies have been retained under constraint.

We defined candidate domesticated retrocopies as those that: (1) are shared only among sloths, (2) are expressed in at least one tissue, (3) encode an ORF at least 70% the length of the parental protein, and (4) have a dN/dS ratio below 0.5. Of 5123 sloth-orthologous retrocopies identified in *C. didactylus*, 2347 show expression, 525 retain a long ORF, and 123 meet all four criteria. Manual inspection of alignments retained 52 high-confidence candidates. As a final quality-control step, we further filtered the RNA-seq data to keep only retrocopies with at least one uniquely mapped read. Fourteen retrocopies were excluded because, although they showed signal in aggregated counts, they showed no uniquely mapped reads to the specific candidate loci (Figure S8). This resulted in a final set of 38 retrocopies with strong evidence of potential domestication.

As an independent validation, we assessed whether the final candidate domesticated set in *C. didactylus* was enriched for evolutionary constraint by comparing dN/dS distributions between candidate loci and other sloth-only retrocopies across all three sloth species. In *C. didactylus*, *C. hoffmanni*, and *B. torquatus*, candidate loci showed significantly lower median dN/dS values than other sloth-only retrocopies (Mann–Whitney tests, *p* < 0.01 in all cases). Candidates were consistently enriched for strong purifying selection (dN/dS < 0.5), with odds ratios ranging from approximately 4 to > 7 across species (Figure S9). Although the strength of constraint varies among individual loci and species—especially for *B. torquatus* where a few loci seem to follow a specific evolutionary trajectory in comparison to *Choloepus*—*the* overall pattern indicates that the candidates are more likely to experience purifying selection than other sloth-only retrocopies. These patterns across species indicate that candidate loci are enriched for evolutionary constraint relative to other sloth-only retrocopies, rather than representing stochastic retention.

**Fig. 6:**
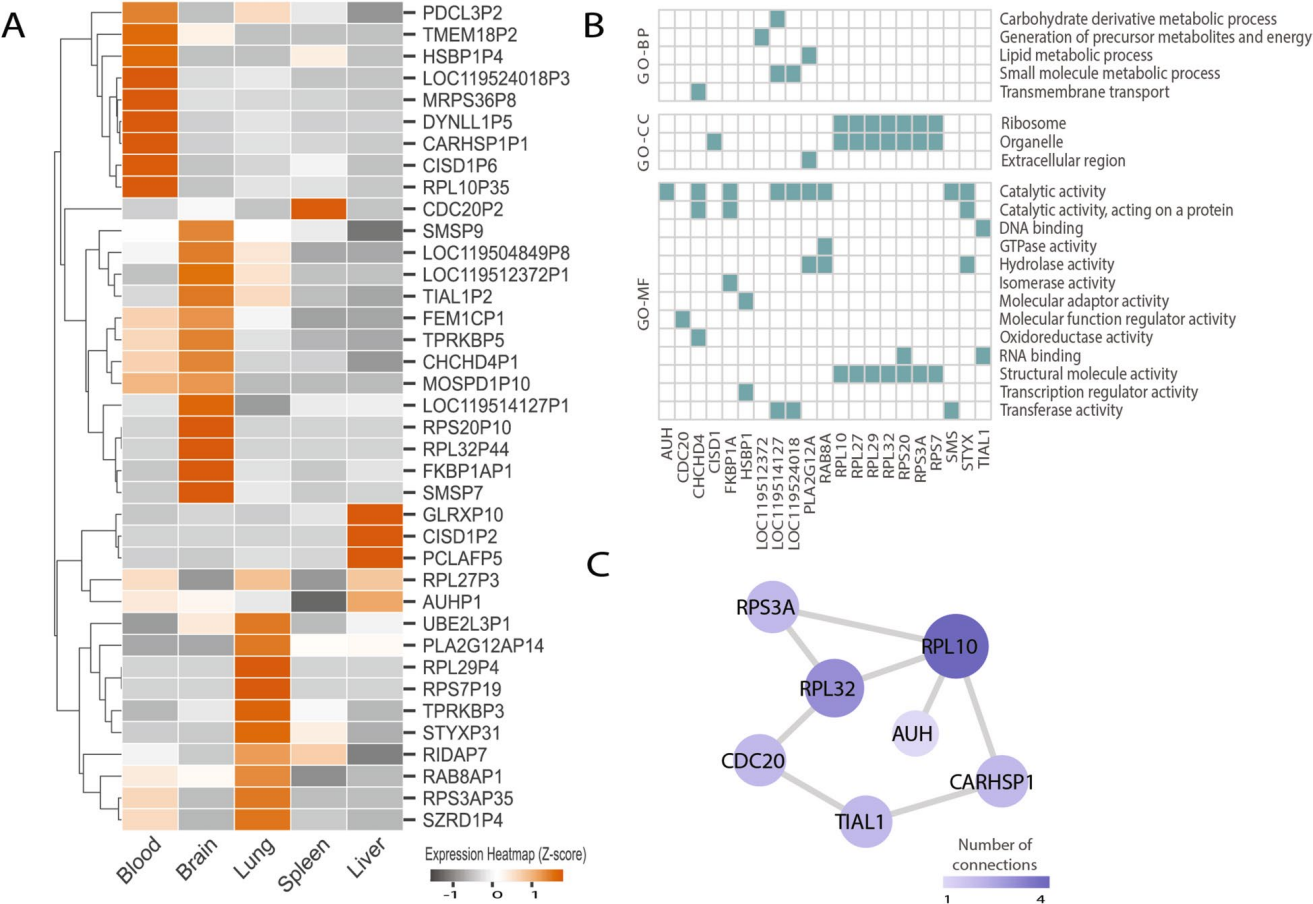
Expression profile and functional characterization of domesticated retrocopy candidates in *C. didactylus*. **A** Heatmap of expression Z-scores normalized per retrocopy across five tissues. Rows represent the retrocopies’ names. Hierarchical clustering was performed using Euclidean distance and complete linkage to highlight expression patterns. **B** Gene Ontology (GO) terms (BP: biological process, MF: molecular function, CC: cellular component) associated with the parental genes depicted in panel **A**. **C** Protein–protein interaction (PPI) network illustrating interactions among the parental genes depicted in panel **A**, highlighting potential functional relationships

The 38 candidate retrocopies represent a core set of genes linked to metabolism, housekeeping, and stress response (Table S6). Many derive from genes involved in mitochondrial, energy metabolism, and lipid regulation (AUH, CHCHD4, CISD1, COX5B, PRPS2-like, MOSPD1, MRPS36, PLA2G12A), stress signalling and heat shock response (HSBP1, CARHSP1), ribosomal proteins (RPL, RPS), and RNA-binding and translation-associated factors (SZRD1, TPRKB). Expression profiles (Fig. 6A) revealed tissue-biased patterns, with many domesticated retrocopies related to mitochondrial and metabolic functions showing elevated expression in the blood, brain, or liver. Stress-response retrocopies were preferentially expressed in blood, while ribosomal retrocopies tended to be either broadly expressed or enriched in specific tissues such as brain or lung. Functional annotation reinforced these categories, with GO terms highlighting roles in ribosome, organelle, catalytic activity, and stress response (Fig. 6B). A protein–protein interaction (PPI) network (STRING, visualized in Cytoscape; Fig. 6C) further underscored connections among mitochondrial, ribosomal, and RNA-binding parental genes.

## Discussion

### New high-quality genomes for xenarthra

Here, we presented two new high-quality genome assemblies for Xenarthra, a placental mammalian clade long underrepresented in genomics. Previous available assemblies for Xenarthra, such as the Sanger-based genome of *Choloepus hoffmanni*, remain highly fragmented despite later Hi-C scaffolding (e.g., 117,974 gaps; contig N50 = 64 Kb). This fragmentation can affect biological interpretation, as illustrated by our analysis of repetitive elements. The apparent scarcity of full-length LINE1 elements in *C. hoffmanni* assembly is most likely a consequence of assembly quality rather than a true biological feature. Our assembly-independent k-mer analysis indicated that young LINE1 sequences remain abundant in this species, even though few full-length elements could be recovered from the assembled genome. Short-read assemblies generated from Sanger and Illumina sequencing are prone to fragmentation and collapse of long repetitive elements such as LINE1s, especially if these elements are recent expansions with high sequence similarity. This is one case that highlights the importance of long-read assemblies for accurately resolving repeat landscapes.

In contrast, our *C. didactylus* assembly reaches a contig N50 of 20 Mb, marking a substantial improvement for Choloepus species. Alongside improved references for *D. novemcinctus* and *B. torquatus*, the genomes of *C. didactylus* and *T. tetradactyla* presented here advance xenarthran genomics and provide a foundation for comparative studies, including the evolution and impact of retrocopies.

### A standardized annotation of retrocopy identification across mammalian genomes

Recently, some of us presented an updated version of RCPedia [19], a comprehensive database cataloguing retrocopies across 44 mammalian genomes. RCPedia offers both an improved identification pipeline and a user-friendly interface for exploring retrocopy data. A significant challenge in retrocopy research is the variability in detection across studies, primarily due to methodological differences, including alignment criteria, filtering thresholds, and parental gene annotation strategies. Such inconsistencies often lead to large discrepancies in reported retrocopy numbers across species, underscoring the need for standardized pipelines in comparative analyses. Our identification pipeline focuses on biological hallmarks of retrotransposition, including tolerance for truncated insertions through a low size threshold, retention of 3′ mRNA sequences, and allowance for post-insertion TE activity within retrocopies. However, some limitations remain, such as the inability to detect mono-exonic parental genes, which can underestimate the final count of retrocopies, cases where the parental gene or reference transcript has been lost, or retrocopies extensively masked by subsequent TE insertions. To ensure robust cross-species comparisons, we applied the same RCPedia pipeline to the newly assembled xenarthran genomes, revealing that the xenarthrans analyzed here harbour the highest number of retrocopies reported for any animal clade to date.

One potential concern in cross-species retrocopy comparisons is the use of different gene annotations, which can influence the pool of parental genes used for retrocopy detection. In our dataset, some genomes rely on RefSeq annotations whereas others use TOGA orthology-based predictions. To assess the impact of annotation source, we reran the retrocopy detection pipeline for *C. didactylus* using both RefSeq and TOGA annotations. This yielded highly concordant results, with 13,110 retrocopies shared between the two datasets, corresponding to 95.55% of TOGA-derived calls and 82.46% of RefSeq-derived calls. This indicates that the vast majority of retrocopies identified using the more conservative TOGA annotation are also recovered using RefSeq.

Because TOGA annotations are based on orthology-driven predictions from conserved mammalian genes, they represent a more conservative gene set and are expected to underestimate lineage-specific retrocopies. Nevertheless, the strong concordance between annotation strategies indicates that the elevated retrocopy burden observed in these xenarthrans is robust to annotation source. For the final analyses, we retained the RefSeq-based dataset for *C. didactylus* and *D. novemcinctus*, as it benefits from transcriptional support and enables detection of a larger number of retrocopies.

### Repeats, retrocopy formation, and evolutionary dynamics

LINE1 activity has long been associated with retrocopy generation in humans and other mammals [11], as the ORF2 protein preferentially binds poly-A tails, unlike other LINEs that recognize more complex motifs [21]. This association is also evident in the xenarthran genomes analyzed here, but the dynamics differ across lineages. Sloths, particularly *C. didactylus*, exhibit an enrichment of young, full-length, and expressed LINE1 families alongside a large number of recent retrocopies. In contrast, armadillo and anteater harbour older LINE1 repertoires, and their clade-specific retrocopies lack signatures of recent activity. Armadillos (Cingulata) are the most diverse xenarthran lineage (25 extant species), followed by anteaters (Vermilingua, eleven species) and sloths (Folivora, seven species) [3]. Our dataset includes both extant sloth genera (*Choloepus* and *Bradypus*), but only a single representative of armadillos and anteaters. Additional high-quality genomes for these lineages will therefore be critical to determine whether the observed retrocopy patterns reflect species-level processes or broader clade-level dynamics.

In mammals, LINE1 expression is typically tightly regulated due to its potential impact on genome stability and cellular homeostasis [22]. In this context, the presence of young and transcriptionally active LINE1 elements in somatic tissues of *C. didactylus*, including blood, might suggest a more permissive landscape of LINE1 expression in sloths compared to most mammals. While the physiological implications of this pattern remain unclear, it is known that LINE1 activity can influence gene expression through multiple mechanisms, including regulatory interference and the generation of novel transcripts [22, 23].

### Retrocopies as material for genomic innovation

The overall retrocopy landscape in Xenarthra mirrors patterns observed in other mammals [19], with a strong enrichment for highly expressed and housekeeping genes. In the tissues we have investigated, about half of retrocopies are transcriptionally active in *C. didactylus*, compared with roughly one third in the armadillo. While a substantial portion of this expression may represent transcriptional noise, retrocopies have also been shown to modulate the expression of their parental genes, acting through mechanisms such as antisense interference, transcript competition, or regulatory disruption [24]. In addition, retrocopies can give rise to entirely novel genes through a process known as retrogene domestication, contributing new genetic material to host genomes [25].

The presence of sloth-only orthologous retrocopies offered an opportunity to look for domesticated retrocopies in this lineage. Sloths are renowned for their exceptionally low metabolic rates—the lowest recorded among mammals—with basal metabolic rates reduced by 69–79% compared to expectations based on body mass [8]. Both *Bradypus* and *Choloepus* exhibit heterothermy, adjusting their internal body temperature in response to ambient temperature [9], displaying a metabolic flexibility rarely seen in mammals. However, the link between this phenotype and the molecular biology of sloths is still not understood. Recent findings from some of us suggest relaxation in the mitochondrial genomes of both sloth genera (Johnson et al., in preparation), potentially reflecting a greater tolerance for mutational accumulation due to their diminished energetic demand. In this study, we identified 38 retrocopies in *C. didactylus* with strong hallmarks of domestication. A substantial fraction of these retrocopies has parental genes functionally linked to mitochondrial processes and cellular metabolism. For example, AUH is a bifunctional mitochondrial protein involved in protein synthesis, RNA metabolism, biogenesis and overall mitochondrial function [26]; CHCHD4 is a central redox-sensitive import factor required for mitochondrial respiratory chain biogenesis [27]; CISD1 is a mitochondrial outer-membrane protein that maintains lipid homeostasis and regulates reactive oxygen species (ROS) production via Fe-S clusters redox reactions [28]; PLA2G12A participates in lipid metabolism [29]; GLRX is a crucial enzyme of the cell’s antioxidant defense system, and may buffer redox imbalance under conditions of compromised electron transport chain (ETC) efficiency; HSBP1 functions as a stress chaperone, potentially stabilizing protein folding under different metabolic profiles. Together, these patterns indicate strong involvement of mitochondrial and metabolic parental genes among domesticated retrocopies, pointing to an intriguing hypothesis: in the face of mitochondrial genome relaxation, some domesticated retrocopies in sloths may act as compensatory buffers, sustaining essential bioenergetic and redox processes, or even providing adaptive routes for metabolic flexibility. Although this hypothesis remains to be experimentally validated—including whether molecular relaxation translates into impaired mitochondrial function in any cellular context—such compensatory roles could be crucial for maintaining baseline metabolic function in an organism adapted to energy conservation. Further functional investigation of these candidates offers a promising direction for understanding genome evolution under conditions of low metabolic demand.

## Conclusions

Xenarthrans possess the highest number of gene duplications mediated by retrotransposition (retrocopies) detected in our comparative dataset, representing the largest retrocopy burden reported among mammals to date. Our results reveal lineage-specific differences in LINE1 activity and retrocopy formation: while activity appears to be older in armadillo and anteater, sloth genomes indicate ongoing LINE1 mobilization in cis (i.e., by retroduplication itself) and in trans (creating retrocopies), and therefore likely a continued impact on their genome architecture. Notably, we identify at least 38 sloth-specific retrocopies with strong signatures of domestication, several of which are functionally linked to metabolism. These may contribute to the distinctive phenotype observed in this highly specialized group of mammals.

## Methods

### Samples, sequencing, and assembly

*Choloepus didactylus* (“Lama Su”) and *Tamandua tetradactyla* (“Anton”) were zoo specimens housed at Tierpark Berlin. Samples were collected post-euthanasia and immediately flash-frozen. For *C. didactylus*, the spleen, blood, brain, lung, and liver were collected. High-molecular-weight DNA was extracted from the spleen for genome sequencing, and all tissues (including spleen) were used for RNA sequencing. For *T. tetradactyla*, spleen tissue was used for DNA sequencing only. All data is available on NCBI under Bioproject Accessions PRJNA678727 and PRJNA561940.

Genome assemblies for both species were generated using the Vertebrate Genomes Project (VGP) pipeline v1.6 [30]. PacBio CLR reads were assembled with FALCON and FALCON-Unzip [31], and haplotigs were removed using Purge-Dups [32]. The primary contigs were scaffolded using 10X Genomics linked reads, Bionano optical maps (Solve), and Hi-C data (Arima) with the SALSA2 pipeline [33]. Assembly polishing was performed in three rounds—first using Arrow with PacBio reads, followed by two rounds with FreeBayes and 10X data [34]. Final decontamination and manual curation were conducted following the methods described in Howe et al. [35]. Assemblies are available on NCBI under accessions GCF_015220235.1 and GCA_023851605.1.

### Genome annotations and other datasets

Short-read RNA sequencing of all *Choloepus didactylus* tissues was performed using a combination of ribosomal RNA depletion (blood) and poly(A) enrichment (spleen, brain, liver, lung). The resulting reads were submitted to NCBI (accession PRJNA516733) and used for genome annotation with the NCBI Eukaryotic Genome Annotation Pipeline. The annotated *C. didactylus* genome is publicly available on NCBI.

Three additional publicly available xenarthran genomes were included in our analyses: *Dasypus novemcinctus* (GCA_030445035.2), annotated via the NCBI pipeline; *Choloepus hoffmanni* (DNA Zoo [36, 37], accession ABVD00000000.2); and *Bradypus torquatus* (GCA_963992745.1). A recent taxonomic review [38] proposed recognizing two maned sloth species: northern (*Bradypus torquatus*) and southern (*B. crinitus*). Under this new classification, the *B. torquatus* used here, which is from a specimen collected in Espírito Santo, Brazil, would represent the *B. crinitus* lineage. For *C. hoffmanni* and *B. torquatus*, we used TOGA annotations [39] provided by Hiller’s lab at the Senckenberg Institute and available at: https://genome.senckenberg.de/download/TOGA/human_hg38_reference/Xenarthra/.

### Repeat annotation, masking, and identification of full-length LINE1 elements

Repeats in the genomes of *Choloepus didactylus*, *Choloepus hoffmanni*, *Bradypus torquatus*, *Tamandua tetradactyla*, and *Dasypus novemcinctus* were identified using EarlGrey [40]. The resulting species-specific repeat libraries were then combined into a single custom library. All five genomes were subsequently masked and annotated with RepeatMasker using this unified repeat library which is available at [41]. Kimura two-parameter substitution levels between each repeat copy and its corresponding consensus sequence were estimated using the calcDivergenceFromAlign.pl script, from RepeatMasker v4.1.5. Repeat landscape plots were generated with custom Python scripts.

To evaluate differences in genomic distribution of young LINE1 insertions, genomes were partitioned into 1 Mb windows and the fraction of bases occupied by LINE1 elements with Kimura divergence ≤ 5% was calculated per window. Differences among species were assessed using Mann–Whitney U tests.

To identify full-length LINE1 retrotransposons, we followed the approach described by Schartl et al. [42]. LINE1 sequences were extracted based on their genomic coordinates, and open reading frames (ORFs) of at least 600 amino acids were identified using the EMBOSS getorf. After further filtering, elements were classified as full-length if they contained two ORFs of ≥ 600 amino acids that spanned at least 90% of both the endonuclease and reverse transcriptase domains.

### Assembly-independent k-mer–based estimation of LINE1 abundance

To assess LINE1 abundance independent of assembly quality, we performed a k-mer–based analysis using raw sequencing reads. Young LINE1 subfamilies were defined as LINE1 annotations with Kimura divergence ≤ 5%. Within each species, subfamilies were ranked by genomic occupancy and those cumulatively accounting for 80% of total young LINE1 sequence were retained. The union of selected families across species comprised 65 subfamilies and was used to define the young LINE1 panel.

Canonical 21-mers were generated from consensus sequences using Jellyfish (v2.3.0) [43], and 50,000 k-mers were randomly sampled per panel. These k-mers were queried against species-specific read k-mer databases (k = 21), and counts were normalized by total read k-mers to obtain counts per million (CPM). The fraction of k-mers detected at least once (hit rate) was also calculated.

### Retrocopy identification pipeline

Retrocopies were identified using our improved pipeline from RCPedia [19]. The approach is based on detecting sequence similarity between multi-exon protein-coding genes (parental genes) and intronless genomic alignments (retrocopies). Messenger RNA (mRNA) sequences were extracted from annotated transcript coordinates using the gffread algorithm [44], and aligned to the genome with the LAST aligner (lastal -D1000) [45] to identify candidate retrocopy insertions. Alignments were retained if they exceeded 120 base pairs in length. To prevent the inclusion of potential chromosomal duplications, candidates located more than 200,000 base pairs from their parental gene were removed. Retrocopies had to preserve at least one of the last three exon-exon boundaries of the parental gene, consistent with the expected reverse transcription mechanism from the poly-A tail. Additionally, alignments containing 40% or more repetitive elements, as identified by RepeatMasker, simpleRepeats, and windowMasker annotations, were excluded. A distance filter restricted retrocopy insertions from the same parental gene to at least 500,000 base pairs apart, reducing false positives without relying on predefined blacklists. Retrocopies overlapping three or more exons of annotated coding genes were excluded, and parental genes with more than five retrocopies overlapping genes from the same gene family were discarded. In cases where multiple alignments originated from the same parental gene, the best-scoring alignment was retained based on sequence identity and match percentage. When ties occurred, the alignment with the highest match/(match + mismatch) ratio was selected. Additionally, continuous alignments from the same mRNA transcript separated by up to 6000 base pairs were merged, accounting for gaps introduced by repetitive elements.

### Retrocopy orthology inferences

To identify potential orthologous retrocopies, we extracted the genomic sequence of each retrocopy along with 3000 base pairs of flanking sequence on both sides. Pairwise alignments were performed using LASTZ [Improved Pairwise Alignment of Genomic DNA, 2007] between each flanked retrocopy region and all corresponding retrocopy regions (including ± 3000 bp flanks) from other genomes, including Bonobo (*Pan paniscus*, GCF_013052645.1), Budgerigar (*Melopsittacus undulatus*, GCF_012275295.1), Cat (*Felis catus*, GCF_000181335.3), Chicken (*Gallus gallus*, GCF_000002315.6), Chimpanzee (*Pan troglodytes*, GCF_002880755.1), Chinese hamster (*Cricetulus griseus*, GCF_003668045.3), Cow (*Bos taurus*, GCF_002263795.1), Crab-eating macaque (*Macaca fascicularis*, GCF_000364345.1), Dog (*Canis lupus familiaris*, GCF_014441545.1), Dolphin (*Tursiops truncatus*, GCF_011762595.1), Drosophila (*Drosophila melanogaster*, GCF_000001215.4), Egyptian rousette (*Rousettus aegyptiacus*, GCF_014176215.1), Gibbon (*Nomascus leucogenys*, GCF_006542625.1), Golden snub-nosed monkey (*Rhinopithecus roxellana*, GCF_007565055.1), Gorilla (*Gorilla gorilla gorilla*, GCF_008122165.1), Greater horseshoe bat (*Rhinolophus ferrumequinum*, GCA_014108255.1), Greater mouse-eared bat (*Myotis myotis*, GCF_014108235.1), Green monkey (*Chlorocebus sabaeus*, GCF_000409795.2), Horse (*Equus caballus*, GCF_002863925.1), Human (*Homo sapiens*, GCF_000001405.39), Kuhl’s pipistrelle (*Pipistrellus kuhlii*, GCF_014108245.1), Lizard (*Anolis carolinensis*, GCF_000090745.1), Marmoset (*Callithrix jacchus*, GCF_009663435.1), Mouse (*Mus musculus*, GCF_000001635.27), Mouse lemur (*Microcebus murinus*, GCF_000165445.2), Opossum (*Monodelphis domestica*, GCF_000002295.2), Orangutan (*Pongo pygmaeus abelii*, GCF_002880775.1), Painted Turtle (*Chrysemys picta bellii*, GCF_000241765.4), Pale spear-nosed bat (*Phyllostomus discolor*, GCA_014049915.1), Panda (*Ailuropoda melanoleuca*, GCF_002007445.1), Pig (*Sus scrofa*, GCF_000003025.6), Platypus (*Ornithorhynchus anatinus*, GCF_004115215.1), Rabbit (*Oryctolagus cuniculus*, GCF_000003625.3), Rat (*Rattus norvegicus*, GCF_000001895.5), Rhesus (*Macaca mulatta*, GCF_003339765.1), Sheep (*Ovis aries*, GCF_002742125.1), Tasmanian Devil (*Sarcophilus harrisii*, GCF_902635505.1), Turkey (*Meleagris gallopavo*, GCF_000146605.3), Velvety free-tailed_bat (*Molossus molossus*, GCF_014108415.1), Zebra Finch (*Taeniopygia guttata*, GCF_008822105.2), Zebrafish (*Danio rerio*, GCF_000002035.6), as previously described [19]. Alignment coverage exceeding 50% and identity surpassing 60% were required. Furthermore, we ensured that at least 50% of the retrocopy aligned with the designated target region. When multiple possible orthologs were identified, the alignment with the highest coverage and identity was selected for each retrocopy. Retrocopy homology was classified into five broad categories: (1) species-specific, referring to retrocopies with no detectable homology in any other species analyzed; (2) sloth-shared, those homologous in two or all sloth species; (3) xenarthra-shared, those with homology in two or more xenarthran species, excluding sloth-shared retrocopies; (4) other-species-shared, comprising retrocopies with homology in at least one non-xenarthran and non-elephant species.

### Genome architecture correlates of retrocopy abundance

To evaluate whether variation in retrocopy counts across species could be explained by differences in genome architecture, we compiled genomic data for 50 species including the 41 mammals, birds, reptiles, amphibians, fish, and insects. For each species, we recorded the total number of retrocopies, the number of annotated protein-coding genes (from the corresponding annotation file), genome size, and the abundance of LINE1s (from Repeat Masker outputs).

We tested for associations between retrocopy counts and three genomic variables: number of protein-coding genes, genome size, and LINE1 abundance. Pairwise relationships were first evaluated using linear regression across species. Because species are not statistically independent due to shared evolutionary history, we repeated these analyses using phylogenetically independent contrasts with ape [46]. The phylogenetic tree used for these analyses was obtained from the TimeTree database (https://timetree.org). Independent contrasts were calculated for each variable and regressions were performed through the origin following standard PIC methodology. Analyses were performed both across the full dataset (50 species) and for a mammal-only subset (41 species) to assess whether patterns were consistent within mammals. For each regression we report slope, *p*-value, and adjusted R2.

### GO annotation and functional enrichment analyses

We used Interproscan to annotate GO terms for parental genes of retrocopies. Later, we matched those GOs with GOslim using goatools map_to_slim.py [47]. Gene Ontology (GO) enrichment analyses of parental genes were performed in R (v. 4.5.1) using the clusterProfiler (v. 4.16.0) package. GO term enrichment was assessed across the three subontologies—biological process (BP), molecular function (MF), and cellular component (CC)—for each species and lineage group (i.e., parental genes of Xenarthra-specific, sloth-specific, and species-specific retrocopies). For each analysis, the background gene set comprised all genes from the corresponding species or lineage group with available GO annotations. Enrichment was computed using the enricher() function, applying a false discovery rate (FDR) cutoff of 0.01 and minimum and maximum gene set sizes of 10 and 500, respectively. Only GO terms with fold enrichment ≥ 2 were retained for downstream interpretation.

### Retrocopies and LINE1 expression quantification in *Choloepus didactylus* and *Dasypus novemcinctus*

To ensure accurate quantification of retrocopy expression despite their high sequence similarity to parental genes, we applied a stringent in silico filtering strategy. We simulated paired-end RNA-Seq data (60 million reads, HiSeq 150 profile) using SANDY [48] v1.0 with protein-coding transcripts as input, explicitly excluding regions overlapping exonic retrocopies (SANDY parameters –sequencing-type paired-end –quality-profile hiseq_150 –number-of-reads 60,000,000 –jobs 20). Quantification was performed with kallisto v0.48.0 [https://pachterlab.github.io/kallisto/about] (-t 12 -b 100). Retrocopies with detectable expression in the simulations (TPM > = 0.1) were deemed potentially confounding and excluded from downstream analysis. Because simulated datasets contained no retrocopy-derived reads, any detected expression represents spurious assignment from parental transcripts, providing an empirical estimate of false-positive signal.

For LINE1s, we simulated reads from consensus sequences in our repeat library. A Kallisto index including both LINE1 consensus sequences and protein-coding transcripts was used for quantification. Only LINE1 subfamilies with a ratio of observed-to-expected simulated expression between 0.8 and 1.15 were retained.

Experimental RNA-Seq data were obtained from five tissues of *C. didactylus*—spleen, brain, liver, lung, and blood (SRR10066840–SRR10066845) and three tissues of *D. novemcinctus* (SRR32745552–SRR32745554)—and quantified using kallisto against a database index built from the following: (i) filtered retrocopies, (ii) simulation-vetted LINE1 consensus sequences, and (iii) RefSeq protein-coding transcripts (Annotation Release 100) excluding retrocopy overlaps.

To avoid ambiguous quantification of closely related retrocopies, particularly those derived from the same parental gene, Choloepus-specific retrocopies sharing a parental origin were grouped as single transcriptional units. Read counts for grouped retrocopies were aggregated using custom scripts. LINE1 expression was measured at the subfamily level and normalized as transcripts per million (TPM).

To validate expression in candidates of domestication in *C. didactylus*, we complemented kallisto quantification with STAR [49] v2.7.7a alignments (–outSAMtype BAM SortedByCoordinate –outSAMattributes NH HI AS nM MD XS), retaining only uniquely mapped reads. Reads aligning to parental genes or other recent duplicates were excluded. STAR-derived values were compared to kallisto estimates to assess consistency.

### dNdS estimation of retrocopies

To estimate nonsynonymous to synonymous substitution rate ratios (dN/dS), we first predicted open reading frames (ORFs) (minimal length = 75aa) in retrocopies using TransDecoder. Predicted amino acid sequences for each retrocopy were aligned to its corresponding parental gene’s protein sequence using ClustalW. Codon-aware nucleotide alignments were then generated with PAL2NAL [50], based on the protein alignments and underlying coding sequences. Synonymous (dS) and nonsynonymous (dN) substitution rates were calculated using codeml from the PAML package [51], using the following parameters: CodonFreq = 2, model = 0, Nsites = 0, fix_omega = 0, and omega = 0.4. Retrocopies with dS ≤ 0.02 were classified as recent insertions, and enrichment of recent retrocopies across species was assessed using Fisher’s exact tests.

### Domestication candidates’ selection

We parsed files from codon-aware alignments, codeML output and RNA-seq expression and defined candidate domesticated retrocopies as those that (1) are shared only among sloths, (2) are expressed in at least one tissue, (3) encode an ORF at least 70% the length of the parental protein, and (4) have a dN/dS ratio below 0.5.

To evaluate whether candidate domesticated retrocopies exhibit lineage-wide evolutionary constraint, we compared dN/dS distributions between candidate loci and other sloth-only retrocopies across *C. didactylus*, *C. hoffmanni*, and *B. torquatus*. The foreground consisted of candidate domesticated retrocopies, whereas the background comprised all remaining sloth-only retrocopies, selecting a single representative ORF per locus defined as the longest predicted ORF. Differences in dN/dS distributions between candidate and background loci were assessed using two-sided Mann–Whitney U tests. Enrichment for purifying selection was evaluated using Fisher’s exact tests with a threshold of dN/dS < 0.5. Effect sizes were quantified using the rank-biserial correlation. To account for potential instability of extreme dN/dS ratios, analyses were repeated after excluding values greater than 5.0.

### Protein–protein interaction analysis

We performed protein–protein interaction (PPI) analysis among parental genes of possibly domesticated retrocopies using Cytoscape (v. 3.10.3) [52], based on interaction data from the STRING database (v. 11.5) [53]. Only interactions with a confidence score greater than 0.15 were retained.

## Supplementary Information

Additional file 1. Manuscript abstract in Portuguese.

Additional file 2. Figure S1: Genome assembly validation for *Choloepus didactylus* and *Tamandua tetradactyla*. Table S1: General metrics of publicly available Xenarthra genomes included in this study. Figure S2: General repeat content of Xenarthra genomes. Table S2: Contiguity comparison of Xenarthra assemblies. Figure S3: Assembly-independent quantification of young LINE1 sequence using k-mer analysis. Table S3: Summary statistics from assembly-independent k-mer quantification of young LINE1 sequence. Figure S4: Correlations between retrocopy counts and genome size, number of protein-coding genes, and LINE1 abundance across vertebrate species present in RCPedia. Table S4: Phylogenetically independent contrasts (PIC) analyses of correlations calculated in Figure S4. Table S5: Statistical analysis of retrocopy burden per parental genes of *C. didactylus* versus other groups. Figure S5: Retrocopy-predicted ORFs features per species. Figure S6: Parental genes and retrocopies expression in *C. didactylus* and *D. novemcinctus*. Figure S7: Distribution of synonymous substitution rates (dS) for expressed retrocopies in *C. didactylus* and *D. novemcinctus*. Figure S8: Expression of domesticated retrocopies in *Choloepus didactylus*. Figure S9: dN/dS distributions for candidate domesticated retrocopies across sloth lineages. Table S6: NCBI-assigned functions to parental genes giving rise to domesticated retrocopies in *C. didactylus*.

## Data Availability

All data for the new genomes are available on NCBI under Bioproject Accessions PRJNA678727, PRJNA848843 and PRJNA489243. Assemblies are available on NCBI under accessions GCF_015220235.1 and GCA_023851605.1. *C. didactylus* RNA-Seq reads can be found under the accession number PRJNA516733. Coordinates of retrocopy predictions can be found at RCPedia (https://www.rcpediadb.org).
