# Supplementary material for: Elevated retrocopy burden and sloth-specific expansions illuminate mammalian genome evolution
Source: BMC Biol. 2026 May 19;24:137. doi: 10.1186/s12915-026-02632-5 (PMC13251070; doi:10.1186/s12915-026-02632-5)
Supplement: Supplementary file 1 — Additional file 1. Manuscript abstract in Portuguese. [file 12915_2026_2632_MOESM1_ESM.docx]

*This translation in* ***Portuguese*** *was submitted by the authors and we reproduce it as supplied. It has not been peer reviewed. Our editorial processes have only been applied to the original abstract in English, which should serve as reference for this article. This translated abstract is published under the same licence as the article.*

**Introdução**: Xenarthras, preguiças, tamanduás e tatus, representam um dos grupos de mamíferos mais especializados do ponto de vista morfológico e fisiológico; no entanto, a base genômica de suas adaptações permanece pouco compreendida. Aqui, apresentamos genomas em nível cromossômico para a preguiça-de-dois-dedos (*Choloepus didactylus*) e o tamanduá-mirim (*Tamandua tetradactyla*), e investigamos como duplicações gênicas mediadas por retrotransposons (retrocópias) moldaram a evolução do genoma nessas e em outras espécies de Xenarthra.

**Resultados**: Análises comparativas revelaram que os genomas de Xenarthras analisados aqui contêm o maior número de retrocópias já reportado entre mamíferos, com dinâmicas de inserção específicas em cada grupo. Os genomas de tamanduás e tatus apresentam repertórios mais antigos de LINE1, bem como inserções de retrocópias antigas e espécie-específicas. Em contraste, os genomas das preguiças exibem uma abundância de LINE1s recentes e também milhares de retrocópias recentes. Além disso, as preguiças compartilham um grande conjunto de retrocópias ortólogas que se originou a partir de um evento evolutivo de retroduplicação no ramo que levou ao seu último ancestral comum (~30 milhões de anos atrás). Em *C. didactylus*, 49% das retrocópias são expressas em cinco tecidos, em comparação com 27% em *Dasypus novemcinctus*, expressas em três tecidos. Análises evolutivas identificaram 38 retrocópias com fortes indícios de domesticação em *C. didactylus*. Muitas dessas retrocópias derivam de genes parentais envolvidos em processos mitocondriais e metabólicos, sugerindo uma possível contribuição genômica para as especializações fisiológicas das preguiças.

**Conclusões**: Em conjunto, nossos achados identificam a retrotransposição como um importante fator na arquitetura genômica dos xenarthras aqui analisados e destacam a formação de retrocópias como um mecanismo para gerar inovação específica em cada linhagem e, possivelmente, especializações biológicas distintas.
