# Supplementary material for: Elevated retrocopy burden and sloth-specific expansions illuminate mammalian genome evolution
Source: BMC Biol. 2026 May 19;24:137. doi: 10.1186/s12915-026-02632-5 (PMC13251070; doi:10.1186/s12915-026-02632-5)
Supplement: Supplementary file 2 — Additional file 2. Figure S1: Genome assembly validation for Choloepus didactylus and Tamandua tetradactyla. Table S1: General metrics of publicly available Xenarthra genomes included in this study. Figure S2: General repeat content of Xenarthra genomes. Table S2: Contiguity comparison of Xenarthra assemblies. Figure S3: Assembly-independent quantification of young LINE1 sequence using k-mer analysis. Table S3: Summary statistics from assembly-independent k-mer quantification of young LINE1 sequence. Figure S4: Correlations between retrocopy counts and genome size, number of protein-coding genes, and LINE1 abundance across vertebrate species present in RCPedia. Table S4: Phylogenetically independent contrasts (PIC) analyses of correlations calculated in Figure S4. Table S5: Statistical analysis of retrocopy burden per parental genes of C. didactylus versus other groups. Figure S5: Retrocopy-predicted ORFs features per species. Figure S6: Parental genes and retrocopies expression in C. didactylus and D. novemcinctus. Figure S7: Distribution of synonymous substitution rates (dS) for expressed retrocopies in C. didactylus and D. novemcinctus. Figure S8: Expression of domesticated retrocopies in Choloepus didactylus. Figure S9: dN/dS distributions for candidate domesticated retrocopies across sloth lineages. Table S6: NCBI-assigned functions to parental genes giving rise to domesticated retrocopies in C. didactylus. [file 12915_2026_2632_MOESM2_ESM.docx]

**Additional files**

Elevated Retrocopy Burden and Sloth-Specific Expansions Illuminate Mammalian Genome Evolution

Marcela Uliano-Silva^1,2,#,*^, Helena Beatriz da Conceição^3#^, Rafael L. V. Mercuri^3,4^,Sylke Winkler^5^, Gabriela D. A. Guardia^3^, Eugene Myers^5^, Shane McCarthy^1^, Alan Tracey^1^, Alexander Suh^6,7^, Mark Blaxter^1^, Pedro A. F. Galante^3,*^, Camila J. Mazzoni^8,9, *^

^1^ Tree of Life, Wellcome Sanger Institute, Cambridge, UK.

^2^ Faculty of Biosciences and Aquaculture, Nord University, Bodø, Norway.

^3^ Molecular Oncology Center, Hospital Sírio Libanês, São Paulo, Brazil.

^4^ The Institute of Mathematics and Statistics of the University of São Paulo, São Paulo, Brazil.

^5^ Max-Planck Institute for Molecular Cell Biology & Genetics, Dresden, Germany

^6^ Centre for Molecular Biodiversity Research, Leibniz Institute for the Analysis of Biodiversity Change, Museum Koenig Bonn, Adenauerallee 160, D-53113 Bonn, Germany

^7^ Bonn Institute for Organismic Biology (BIOB) – Animal Biodiversity, University of Bonn, Bonn, Germany

^8^ Department of Evolutionary Genetics, Leibniz Institute for Zoo and Wildlife Research (IZW), Berlin, Germany.

^9^ Berlin Center for Genomics in Biodiversity Research (Begendiv), Berlin, Germany.

*Corresponding authors: Email: [mu2@sanger.ac.uk](mailto:mu2@sanger.ac.uk) (M.U.S), [pgalante@mochsl.org.br](mailto:pgalante@mochsl.org.br) (P.A.F.G.), [mazzoni@izw-berlin.de](mailto:mazzoni@izw-berlin.de) (C.J.M.)

#Equal contributors

**Supplementary Figures**


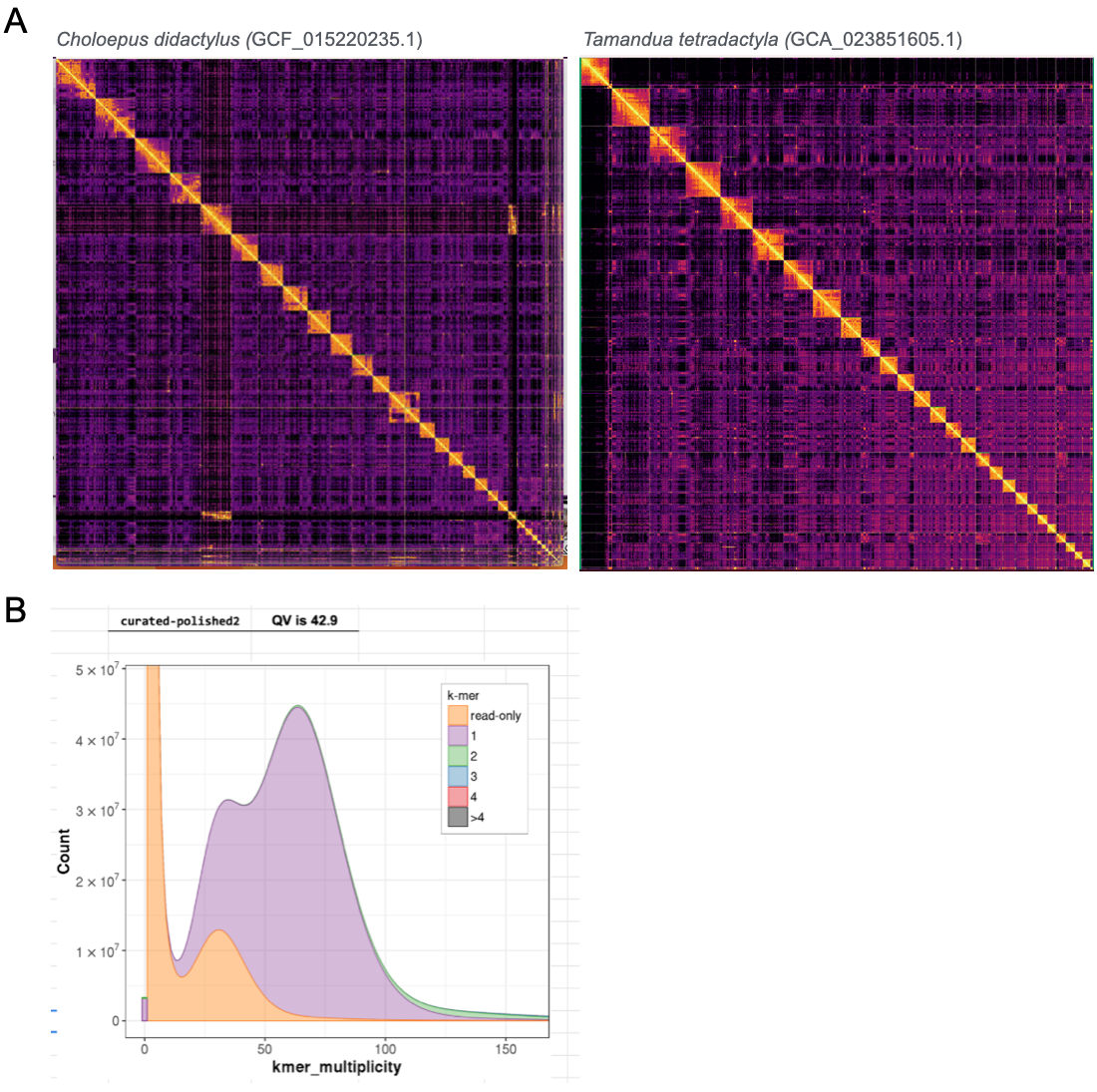


**Figure S1. Genome assembly validation for Choloepus didactylus and Tamandua tetradactyla.** A) Hi-C heatmaps for Choloepus didactylus and Tamandua tetradactyla. B) Merqury plot for C. didactylus final assembly: kmer counts show low levels of duplicated kmers in homozygous and heterozygous genome coverage peaks and distribution.


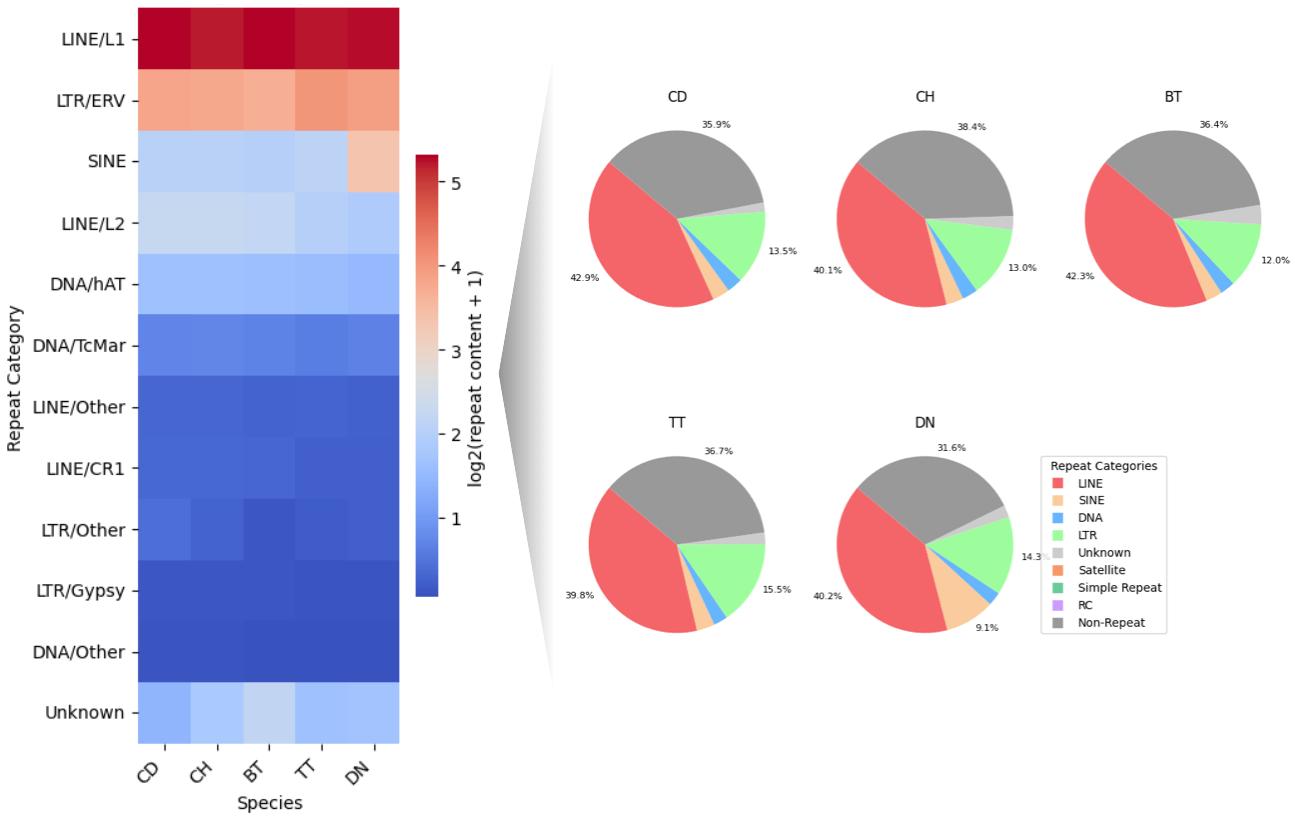


**Figure S2**: General repeat content of Xenarthra genomes. Species abbreviations: CD, *C. didactylus*; CH, *C. hoffmanni*; BT, *B. torquatus*; TT, *T. tetradactyla*; DN, *D. novemcinctus*.

**
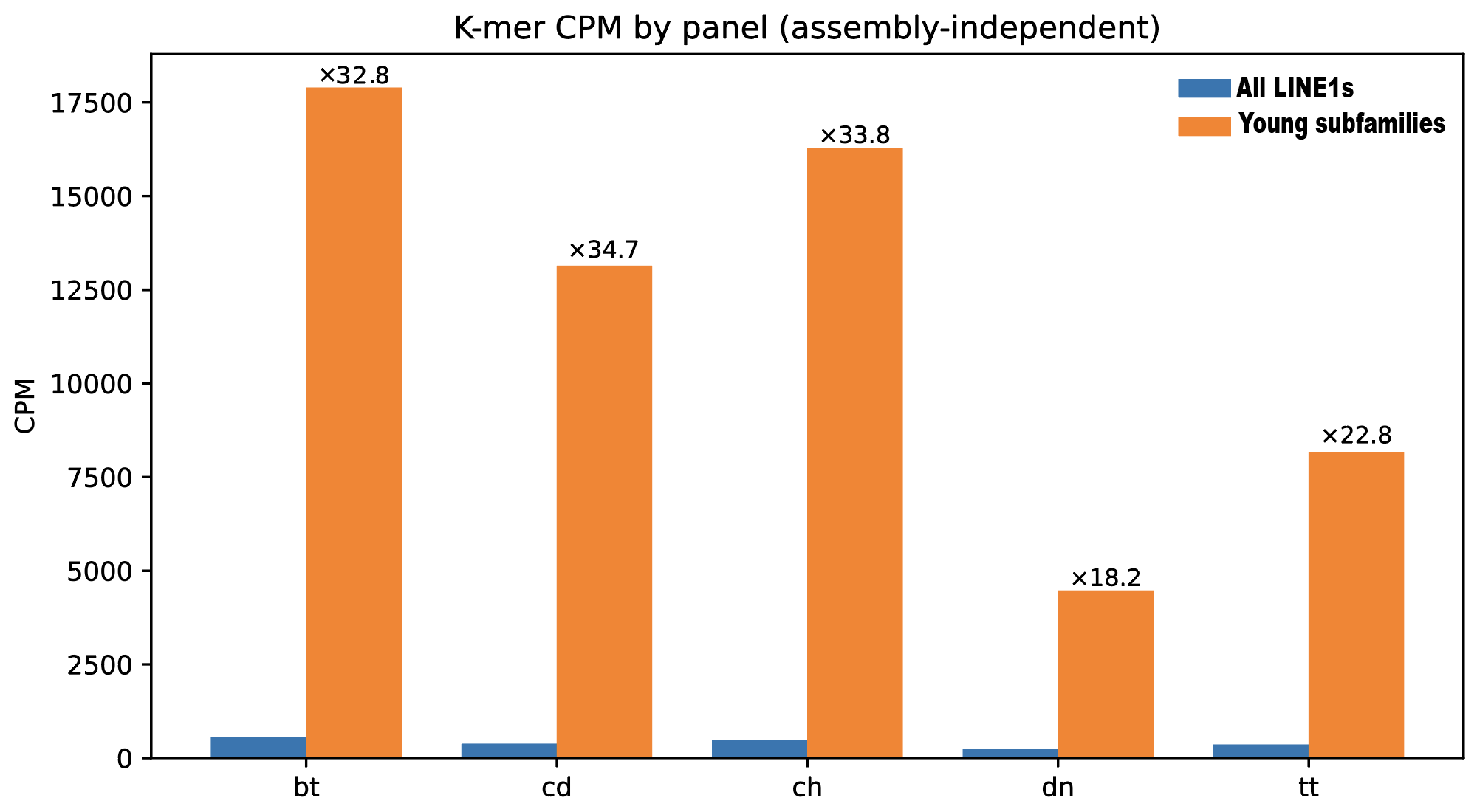
**

**Figure S3: Assembly-independent quantification of young LINE1 sequence using k-mer analysis.** Counts per million (CPM) of k-mers derived from young LINE1 subfamilies were quantified directly from raw sequencing reads for each species. Two reference sets were used: (i) all xenarthran LINE1 subfamilies present in the repeat library and (ii) a curated set of 65 LINE1 sequences (“young subfamilies”). Within each species, LINE1 subfamilies were ranked by genomic occupancy, and those cumulatively accounting for 80% of the total young LINE1 sequence were retained for the curated set. Bars represent normalized k-mer counts (CPM) detected in sequencing reads. Values above the bars indicate the enrichment ratio of the curated young set relative to the full set of xenarthran LINE1 subfamilies for each species. Species abbreviations: CD, *Choloepus didactylus*; CH, *Choloepus hoffmanni*; BT, *Bradypus torquatus*; TT, *Tamandua tetradactyla*; DN, *Dasypus novemcinctus*.


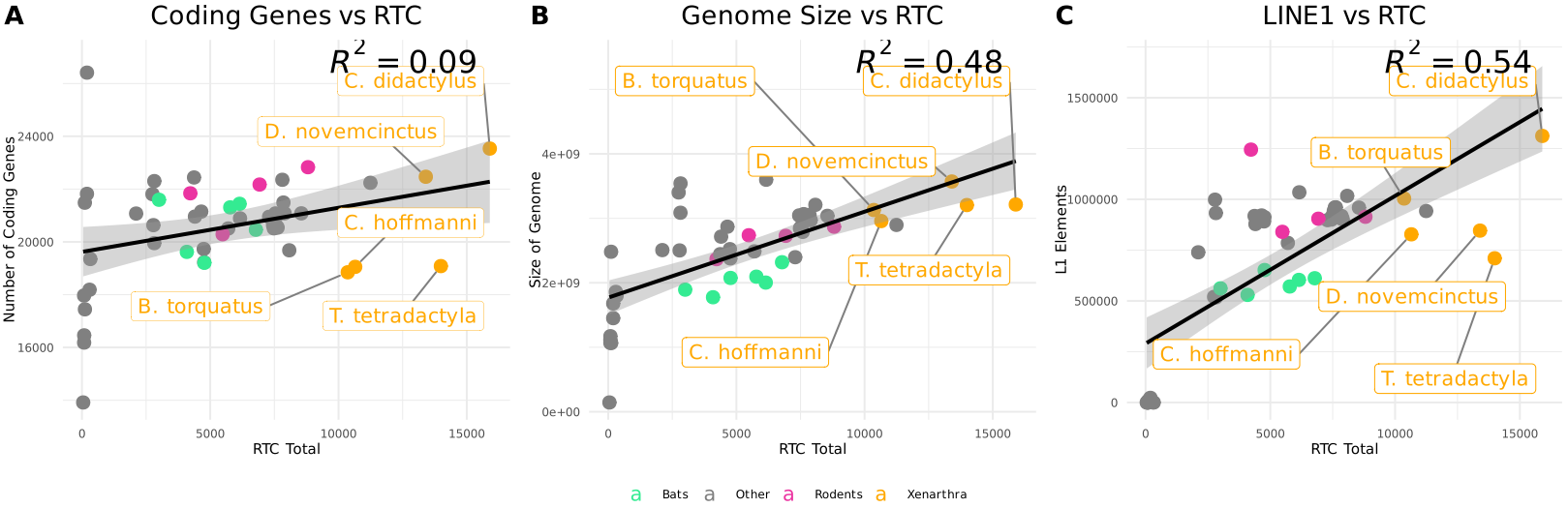


**Figure S4**: Correlations between retrocopy counts and genome size, number of protein-coding genes, and LINE-1 abundance across vertebrate species present in RCPedia. Xenarthra genomes are indicated in orange, rodents in pink and bats in green.


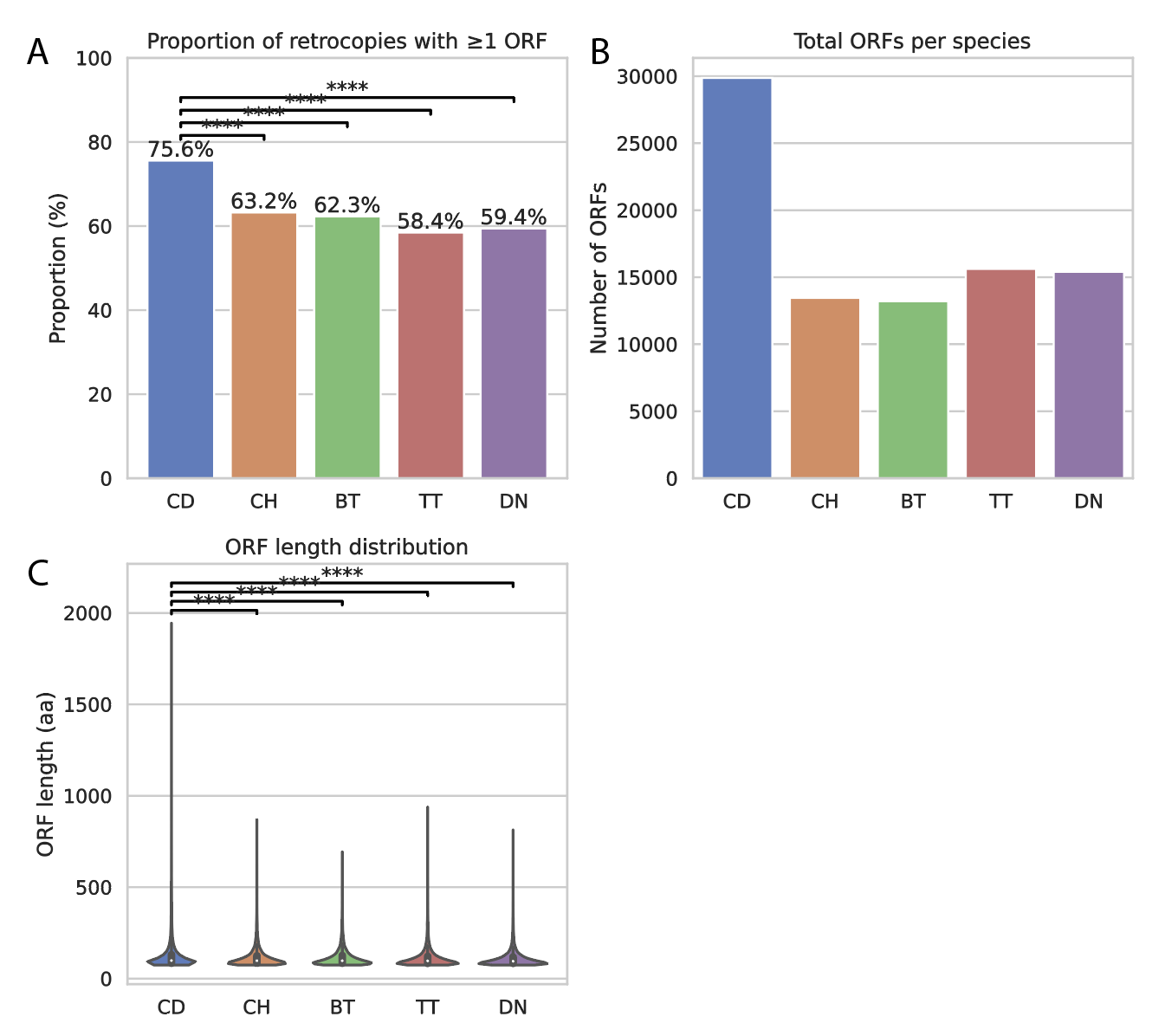


**Figure S5**: Retrocopy-predicted ORFs. A) Proportion of retrocopies yielding ORFs, Significance indicates pairwise comparisons between *C. didactylus* and the other species using two-proportion z-tests (**, p < 0.0001).** B) Total predicted ORFs per species, C) Length of retrocopy-predicted ORFs. Significance indicates pairwise Wilcoxon rank-sum tests comparing *C. didactylus* with other species (, p < 0.0001). Species abbreviations: CD, *C. didactylus*; CH, *C. hoffmanni*; BT, *B. torquatus*; TT, *T. tetradactyla*; DN, *D. novemcinctus*.


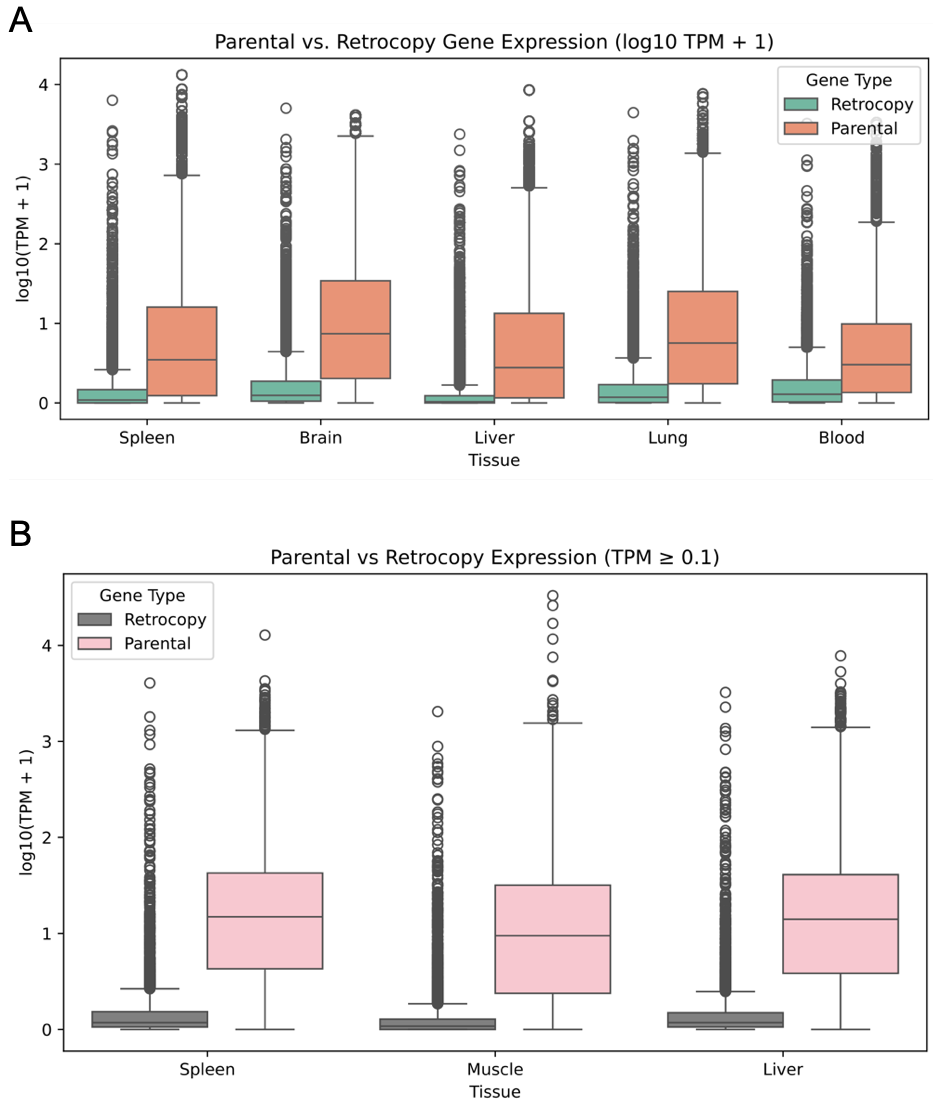


**Figure S6**. Parental genes and retrocopies expression in: A) *C. didactylus*; B) *D. novemcinctus.*


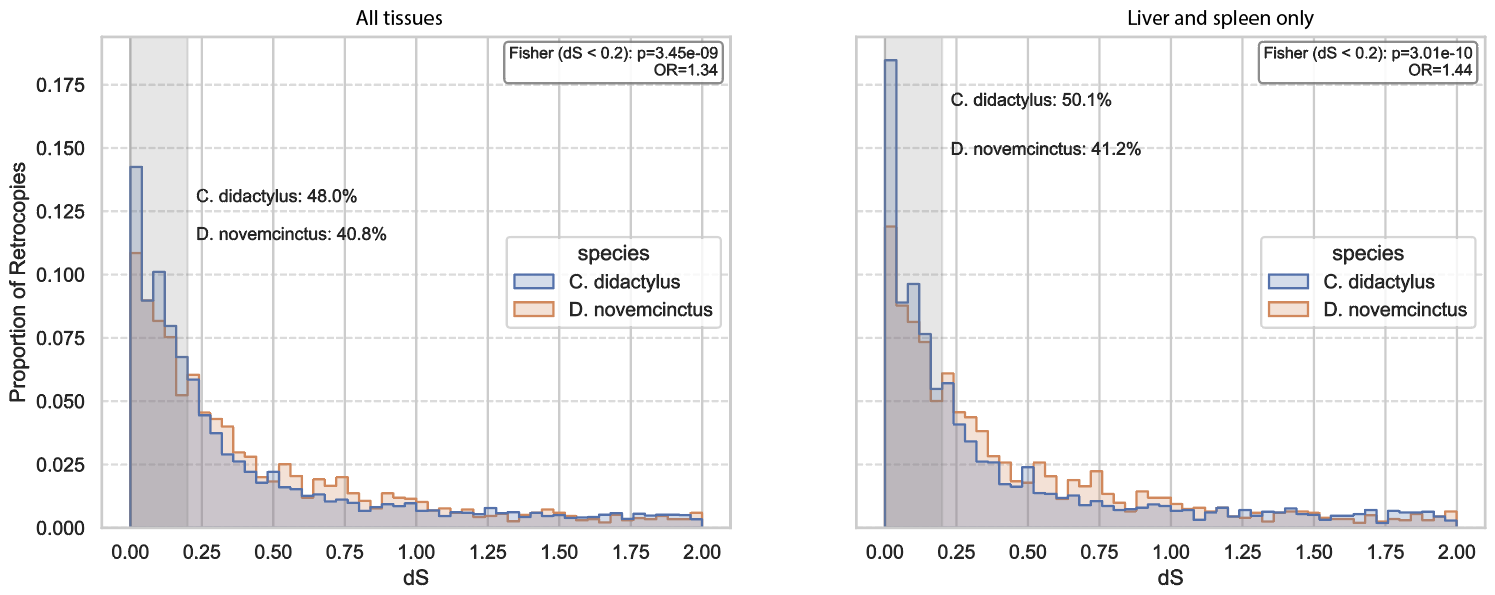


**Figure S7. Distribution of synonymous substitution rates (dS) for expressed retrocopies in *C. didactylus* and *D. novemcinctus***. The overlay histogram shows the proportion of expressed retrocopies across dS bins, with the shaded region highlighting young retrocopies (dS < 0.2). The left panel shows the full dataset using all available tissues, while the right panel shows a controlled analysis restricted to shared tissues (liver and spleen). In both cases, a higher proportion of expressed retrocopies in *C. didactylus* fall within the low-dS range compared to *D. novemcinctus* (full dataset: Fisher’s exact test, p = 3.4 × 10⁻⁹, OR = 1.34; shared tissues: p = 3.0 × 10⁻¹⁰, OR = 1.44), indicating that the enrichment of young expressed retrocopies in *C. didactylus* is robust to differences in tissue sampling.


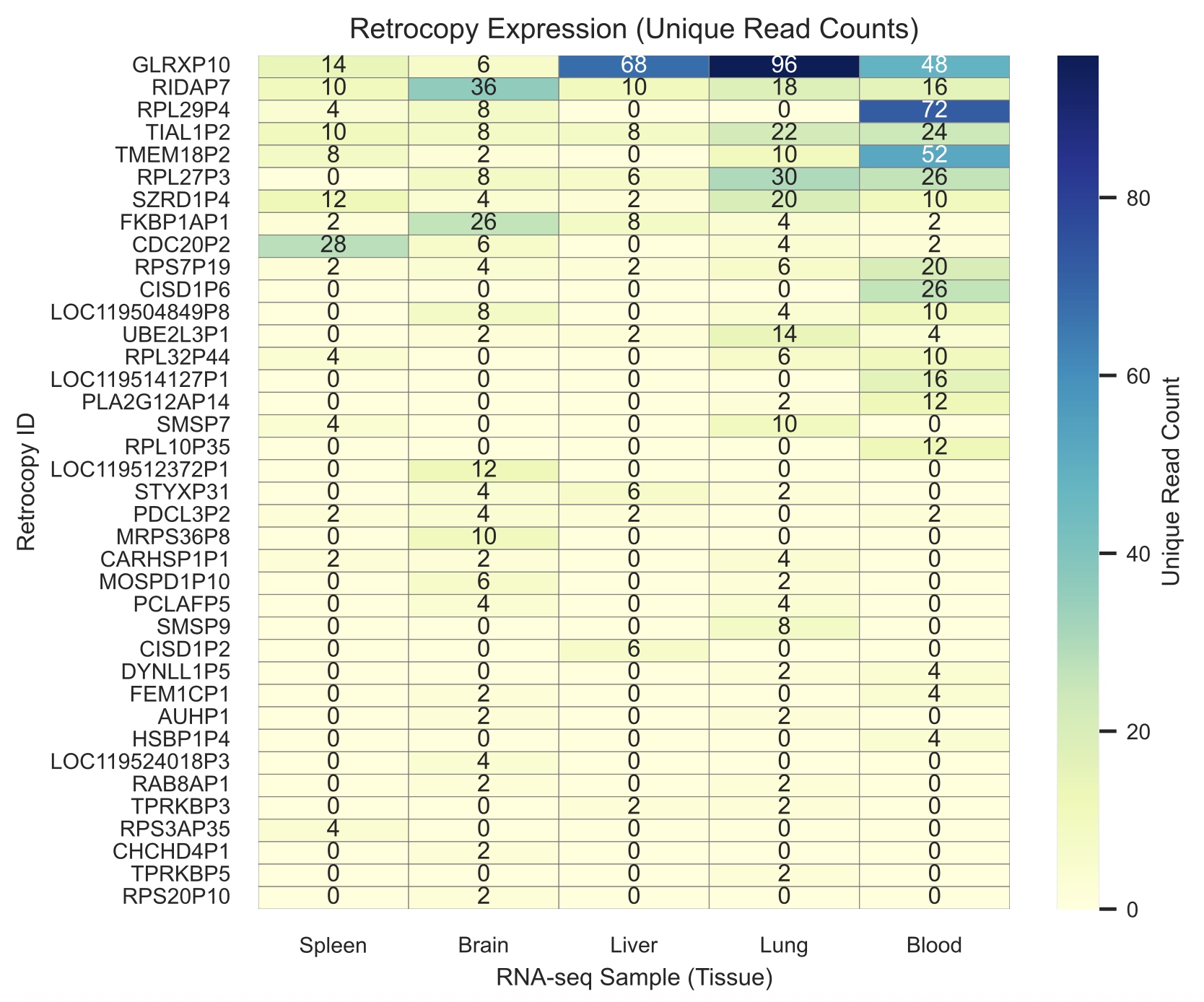


**Figure S8. Expression of domesticated retrocopies in *Choloepus didactylus*.** Expression levels were estimated using uniquely mapped RNA-seq reads.

**
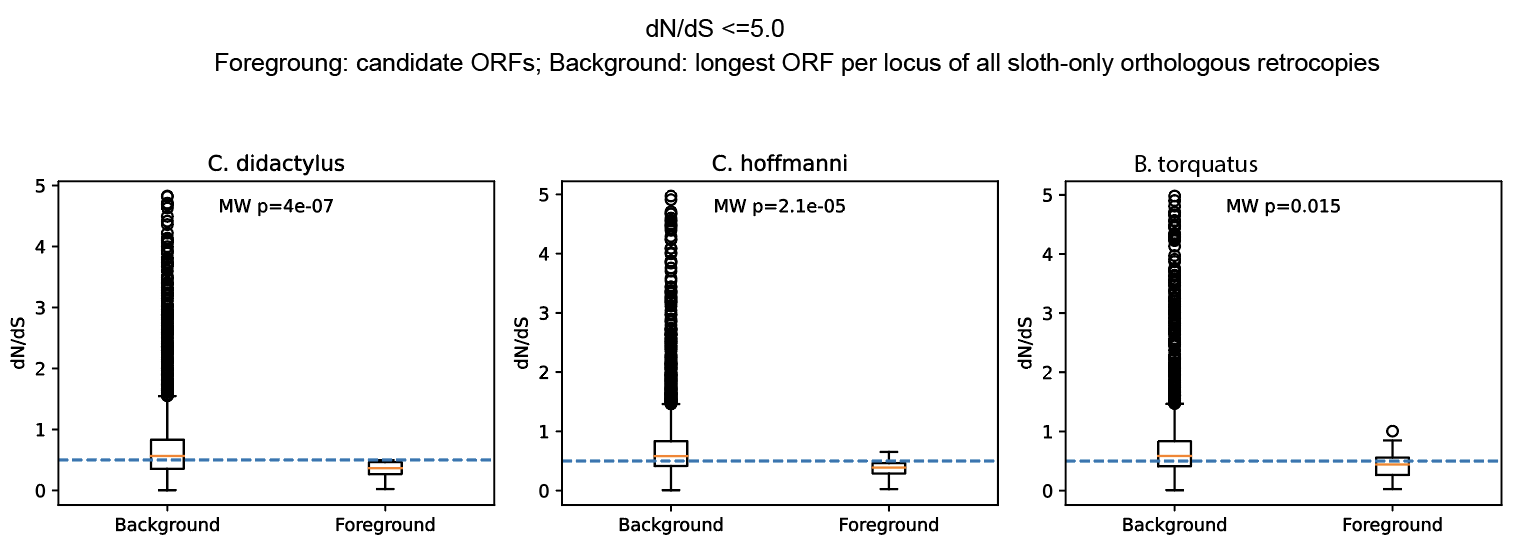
**

**Figure S9. dN/dS distributions for candidate domesticated retrocopies across sloth lineages.** Boxplots show dN/dS distributions for candidate retrocopies (foreground) and remaining sloth-only retrocopies (background) in *C. didactylus*, *C. hoffmanni*, and *B. torquatus*, after excluding extreme values (dN/dS > 5.0) to account for potential ratio instability. The background includes one representative ORF per locus (longest predicted ORF). Dashed horizontal lines indicate the purifying selection threshold (dN/dS = 0.5). P-values from two-sided Mann–Whitney U tests are shown above each panel. Candidate loci exhibit significantly lower dN/dS values than sloth-only background in all three species.

**Supplementary Tables**

**Table S1. General metrics of publicly available Xenarthra genomes included in this study.**

| Species | *Choloepus hoffmanni* | *Bradypus torquatus* | *Dasypus novemcinctus* |
| --- | --- | --- | --- |
| Accession | DNAzoo  C_hoffmanni-2.0.1_HiC.fasta.gz | GCA_963992745.1 | GCF_030445035.2 |
| Total length | 3,293,892,468 | 3,128,591,653 | 3,610,551,914 |
| Scaffold N50 | 140,950,122 | 156,600,181 | 127,081,865 |
| Contig N50 | 64,321 | 4,748,776 | 13,963,394 |
| Scaffold count* | 253,016 | 2,915 | 546 |
| BUSCO** mammalian_odb10 | C:94.2%[S:89.7%,D:4.5%],F:1.9%,M:3.9%,n:9226 | C:94.4%[S:90.1%,D:4.3%],F:1.1%,M:4.5%,n:9226 | C:93.8%[S:90.6%,D:3.2%],F:1.7%,M:4.5%,n:9226 |

*In cases where identified, Including the mitochondrial genome as a scaffold

**BUSCO: Benchmarking using single-copy orthologues

**Table S2. Contiguity comparison (non-N contigs within scaffolds of Xenarthra assemblies)**

| Species | Number of contigs | Max contig length | % non-N bp ≥10 kb | % non-N bp ≥20 kb |
| --- | --- | --- | --- | --- |
| Choloepus hoffmanni | 370998 | 0.71 Mb | 87.01% | 79.47% |
| Choloepus didactylus | 1386 | 67.7 Mb | 99.99% | 99.97% |
| Bradypus torquatus | 5022 | 30 Mb | 99.89% | 99.59% |
| Dasypus novemcinctus | 1339 | 82.5 Mb | 100% | 99.99% |
| Tamandua tetradactyla | 1162 | 46.6 Mb | 99.98% | 99.96% |

**Table S3 | Summary statistics from assembly-independent k-mer quantification of young LINE1 sequence.**

Counts per million (CPM) of k-mers derived from LINE1 sequences were quantified directly from raw sequencing reads for each species. Two reference sets were used: (i) *Young subfamilies*, corresponding to the subset of LINE1 subfamilies with ≤5% Kimura divergence that cumulatively account for ~80% of young LINE1 genomic occupancy within each species, and (ii) *All LINE1s*, representing the full set of xenarthran LINE1 subfamilies identified in the repeat library. For each reference set, 50,000 representative k-mers were queried against raw sequencing reads. Columns report the number of query k-mers (num_kmers), the total number of matching k-mer counts in the reads (sum_counts), the number of k-mers detected at least once (num_present), the fraction of detected k-mers (hit_rate), the total number of k-mers in the sequencing dataset (total_kmers), the fraction of reads matching the query set (fraction), and normalized counts per million (CPM). Abbreviations: CD, *Choloepus didactylus*; CH, *Choloepus hoffmanni*; BT, *Bradypus torquatus*; TT, *Tamandua tetradactyla*; DN, *Dasypus novemcinctus*.

| Species | LINE sequences | num_kmers | sum_counts | num_present | hit_rate | total_kmers | fraction | CPM |
| --- | --- | --- | --- | --- | --- | --- | --- | --- |
| TT | Young subfamilies | 50000 | 217570456 | 19519 | 0.39038 | 26637966290 | 0.008168 | 8167.68 |
| DN | Young subfamilies | 50000 | 78980822 | 13552 | 0.27104 | 17662279281 | 0.004472 | 4471.72 |
| BT | Young subfamilies | 50000 | 612090161 | 35888 | 0.71776 | 34205970582 | 0.017894 | 17894.25 |
| CD | Young subfamilies | 50000 | 453673836 | 36188 | 0.72376 | 34528976896 | 0.013139 | 13138.93 |
| CH | Young subfamilies | 50000 | 58333292 | 34520 | 0.6904 | 3585115419 | 0.016271 | 16270.97 |
|  |  |  |  |  |  |  |  |  |
| TT | All LINE1s | 50000 | 9536040 | 8236 | 0.16472 | 26637966290 | 0.000358 | 357.99 |
| DN | All LINE1s | 50000 | 4337677 | 7203 | 0.14406 | 17662279281 | 0.000246 | 245.59 |
| BT | All LINE1s | 50000 | 18689122 | 8914 | 0.17828 | 34205970582 | 0.000546 | 546.37 |
| CD | All LINE1s | 50000 | 13077273 | 10103 | 0.20206 | 34528976896 | 0.000379 | 378.73 |
| CH | All LINE1s | 50000 | 1726944 | 7981 | 0.15962 | 3585115419 | 0.000482 | 481.70 |

**Table S4: Phylogenetically independent contrasts (PIC) analyses of correlations calculated in Figure S4. Phylogenetic tree recovered from** [**https://timetree.org/**](https://timetree.org/) **for the species used in this study.**

| dataset | predictor | slope | p_value | adj_r_squared | n_species | n_contrasts |
| --- | --- | --- | --- | --- | --- | --- |
| all | coding | 0.3989 | 0.0001 | 0.2561 | 50 | 49 |
| all | genome | 61443.1728 | 0.0002 | 0.2442 | 50 | 49 |
| all | repeats | 42.8247 | 0.0001 | 0.2737 | 50 | 49 |
| mammals_only | coding | 0.4051 | 0.0002 | 0.2801 | 41 | 40 |
| mammals_only | genome | 58399.0253 | 0.0004 | 0.2579 | 41 | 40 |
| mammals_only | repeats | 41.2432 | 0.0004 | 0.2593 | 41 | 40 |

**Table S5: Statistical analysis of retrocopy burden per parental genes of *C. didactylus* versus other groups.**

| Specie 1 | Specie 2 | *p* | *p-adj* | *p.format* | *p.sign* | Method |
| --- | --- | --- | --- | --- | --- | --- |
| *C.didactylus* | Primates | 1.03E-47 | 3.70E-47 | <2e-16 | **** | Wilcoxon |
| *C.didactylus* | Rodents | 4.95E-34 | 1.40E-33 | <2e-16 | **** | Wilcoxon |
| *C.didactylus* | Mammals | 2.07E-109 | 7.50E-108 | <2e-16 | **** | Wilcoxon |
| *C.didactylus* | Others | 1.55E-78 | 1.90E-77 | <2e-16 | **** | Wilcoxon |
| *C.didactylus* | *T.tetradactyla* | 6.40E-11 | 9.60E-11 | 6.40E-11 | **** | Wilcoxon |
| *C.didactylus* | *D.novemcinctus* | 0.1142628717 | 0.12 | 0.1143 | ns | Wilcoxon |
| *C.didactylus* | *C.hoffmanni* | 3.16E-18 | 5.70E-18 | <2e-16 | **** | Wilcoxon |
| *C.didactylus* | *B.torquatus* | 9.85E-22 | 2.00E-21 | <2e-16 | **** | Wilcoxon |

**Table S6**: NCBI-assigned functions to parental genes given rise to domesticated retrocopies in *C. didactylus*

**
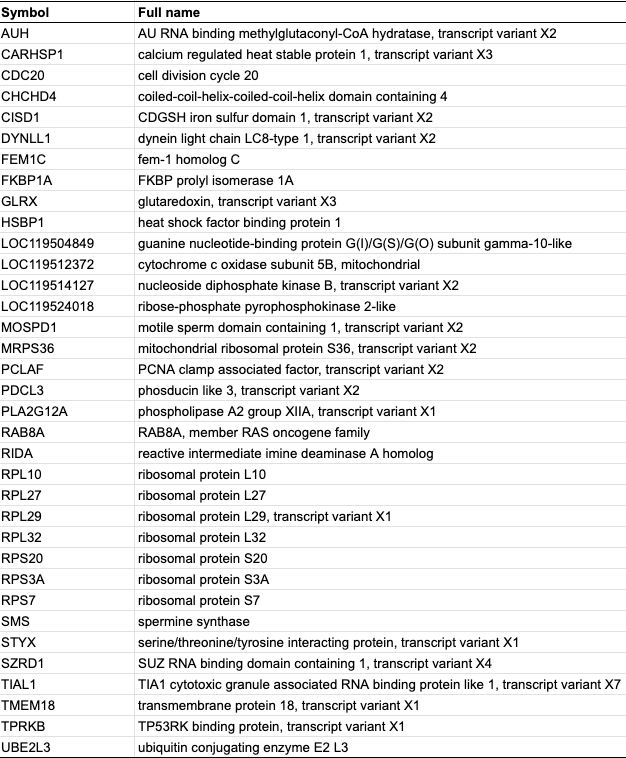
**
